# Homer condensates orchestrate YAP–Wnt signaling crosstalk downstream of the Crumbs polarity complex

**DOI:** 10.1073/pnas.2523230123

**Published:** 2026-07-15

**Authors:** Siti Maryam J. M. Yatim, Linda Jiabao Woo, Yuhong Chen, Barbara Hübner, Alexander Ludwig

**Affiliations:** ^a^ School of Biological Sciences, Nanyang Technological University, Singapore 637551, Singapore; ^b^ Nanyang Technological University (NTU) Institute of Structural Biology, Nanyang Technological University, Singapore 636921, Singapore

**Keywords:** crumbs complex, YAP, homer, wnt signaling, phase separation

## Abstract

The Hippo pathway governs cell growth, proliferation, and differentiation and is frequently deregulated in cancer. Yes-associated protein (YAP) is the central transcriptional coactivator of the Hippo pathway and interacts with β-catenin to coordinate YAP–Wnt signaling crosstalk. Both pathways are modulated by diverse upstream signals including mechanical cues, cell density, and cell polarity, yet how such signals are integrated remains poorly understood. Here, we demonstrate that Homer scaffolding proteins coordinate YAP and Wnt signaling downstream of the Crumbs polarity complex. Homers interact directly via their EVH1 domains with the Crumbs component PATJ and the NDR kinase scaffold Furry-like (FRYL). Homers antagonize FRYL to promote YAP activation while cooperating with FRYL to enhance Wnt/β-catenin signaling, revealing pathway-selective regulation. PATJ, in contrast, recruits Homers to the cortex and restrains YAP activity. We further show that Homers form biomolecular condensates in nonpolarized epithelial and colorectal cancer cells, whose assembly and signaling properties are differentially modulated by PATJ and FRYL. Whereas FRYL promotes the formation of cytoplasmic droplets, PATJ drives the assembly of phase-separated compartments at or near the plasma membrane. Collectively, our findings establish Homer-driven phase separation as a tunable signaling mechanism to translate polarity cues into transcriptional output.

## Significance

Cell polarity complexes are fundamental regulators of tissue growth, yet the molecular mechanisms linking polarity cues to transcriptional control remain poorly understood. Here, we identify Homer proteins as condensate-forming effectors of the Crumbs polarity complex that orchestrate Yes-associated protein (YAP)–Wnt signaling crosstalk in epithelial and cancer cells. We show that Homer condensates are tuned by paralogue identity, protein abundance, and binding partners, revealing how changes in phase behavior shape signaling output. Our findings establish biomolecular condensation as a key organizing principle for YAP–Wnt pathway coordination and provide insight into how the spatial organization of signaling networks governs cell behavior and tissue homeostasis.

The Hippo pathway is a central regulator of cell growth, proliferation, differentiation, and survival, and is frequently deregulated in cancer. Its core effectors are the transcriptional coactivators Yes-associated protein (YAP) and its paralogue TAZ (WWTR1), which shuttle between the cytoplasm and nucleus to control gene expression. In the canonical Hippo pathway, the Ste20-like kinases MST1/2 activate the Large Tumor Suppressor kinases LATS1/2, which phosphorylate YAP/TAZ on multiple serine/threonine residues. This promotes cytoplasmic retention or proteasomal degradation, thereby preventing YAP/TAZ from activating TEA domain transcription factors (TEADs) in the nucleus (1, 2). Several noncanonical kinases—including MST3/4, NDR1/2, MAP4Ks, and MEKK family members—also modulate YAP/TAZ (2–6). Recent studies further indicate that biomolecular condensation of Hippo components contributes to pathway regulation. Such phase separated assemblies are modulated by diverse upstream inputs, including osmotic stress, cytoskeletal tension, extracellular matrix stiffness, cell–cell junctions, and polarity cues (7–14). However, how the assembly and signaling output of Hippo condensates is regulated is poorly understood.

YAP/TAZ also engage in extensive crosstalk with the TGFβ, NOTCH, and Wnt/β-catenin signaling pathways (15–24). In the absence of a Wnt signal, cytoplasmic YAP/TAZ are incorporated into the β-catenin destruction complex via an interaction with Axin1 and promote β-catenin degradation (21). When Wnt signaling is activated, YAP/TAZ dissociate from the destruction complex and translocate into the nucleus to form transcriptional complexes with β-catenin and the TCF/LEF transcription factors, leading to the expression of YAP/TAZ and Wnt-dependent target genes (21, 24). YAP/TAZ may also suppress Wnt signaling by binding to Disheveled, which prevents its activation (20), or by binding directly to β-catenin, which stabilizes β-catenin in the cytoplasm (23). Moreover, both canonical and noncanonical Wnt signaling can feedback to the Hippo pathway to control the activity of YAP (25, 26). Such complex reciprocal interactions between the Hippo and Wnt pathways have significant implications during differentiation, regeneration, and in cancers (27–29), yet how this pathway crosstalk is coordinated remains largely unclear.

The epithelial Crumbs complex, which is composed of the transmembrane protein Crumbs (Crb), the adaptor protein Pals1 (Protein Associated with Lin seven 1), and the multi-PDZ domain scaffolding protein PATJ (Pals1-Associated Tight Junction protein), is an evolutionarily conserved regulator of cell polarity and an important upstream regulator of the Hippo pathway (30–34). In *Drosophila*, Crb restricts tissue growth by promoting apical retention and inhibition of Yorkie (the YAP/TAZ orthologue) (35–38). Similarly, loss of Crb3 or Pals1 in mammalian cells and mouse models leads to YAP/TAZ hyperactivation, accompanied by defects in tissue architecture and differentiation (39–42). Despite these observations, the molecular mechanisms linking the Crumbs complex to Hippo/YAP regulation—particularly in mammalian epithelial cells—remain incompletely defined.

We previously demonstrated that the mammalian Crumbs complex defines a distinct cortical domain apical of epithelial tight junctions, termed the vertebrate marginal zone (VMZ) (32, 43). In that study we identified the Homer scaffolding proteins as novel PATJ interactors. Homers (Homer1 to 3) are composed of an N-terminal EVH1 domain that binds PPxxF motif–containing ligands and a C-terminal coiled coil domain that mediates homo- and heterotetramerization (44–46). This tetrameric organization confers multivalency, a key feature underlying their ability to assemble biomolecular condensates with postsynaptic density proteins (47–49). Beyond their established roles in synaptic signaling and actin bundling (47, 50), emerging evidence implicates Homers in calcium-dependent mechanosensing at epithelial cell junctions (51), neutrophil cell polarity and migration (52), and YAP and β-catenin signaling in cancer cells (53–57). Here, we report that Homers promote YAP/TEAD and canonical Wnt signaling in polarized and nonpolarized epithelial cells through the formation of biomolecular condensates that are differentially regulated by PATJ and the NDR kinase scaffolding protein FRYL.

## Results

### PATJ Directly Interacts with and Recruits Homers to Apical Cell Junctions.

**Fig. 1.**
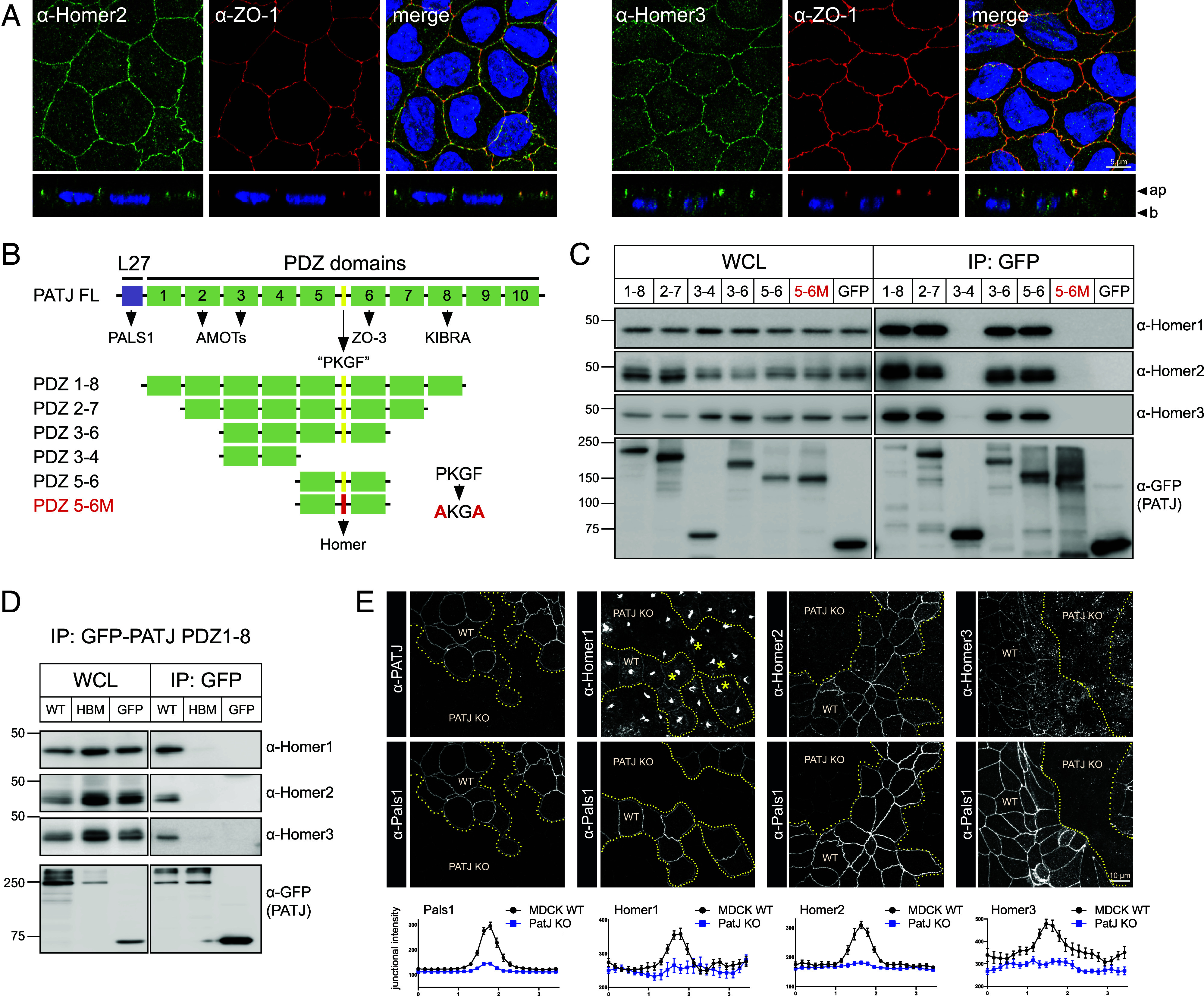
Homers are recruited to apical cell junctions via a direct interaction with PATJ (*A*) Airyscan microscopy of endogenous Homers in MDCK-II cells. Cells were grown to confluency on Transwell filters for 14 d, fixed and costained with ZO-1 and Homer2 or Homer3 antibodies. XY (en face) and XZ (side view) projections are shown. (*B*) Domain organization of PATJ and representation of PATJ PDZ domain constructs. Known binding partners of PATJ and the Homer binding site (PKGF) are indicated. (*C*) Coimmunoprecipitation (Co-IP) assay in 293 T cells transfected with GFP-tagged PATJ constructs shown in (*B*). The interaction with endogenous Homer proteins was analyzed by western blotting (WB). (*D*) Co-IP assay of GFP-PATJ PDZ1-8 and the corresponding Homer binding mutant. (*E*) Maximum intensity projections of confocal z-stacks of MDCK wild-type (WT) and PATJ KO cells cocultured on Transwell filters for 12 d. Pals1 antibody served as a marker for WT cells. Note that anti-Homer1 antibodies produce a nonspecific staining of unknown origin (yellow asterisks). *Bottom*: Average junctional signal intensity of Pals1 and Homers in PATJ KO and MDCK WT cells determined by line scan analysis (n = 11 each).

We demonstrated previously that GFP-tagged Homers localize to apical cell junctions in fully polarized Madin Darby Canine Kidney type II (MDCK-II) cells (43). Consistent with this, immunofluorescence analysis of fully polarized MDCK monolayers demonstrated that endogenous Homer1, Homer2, and Homer3 colocalize with the tight junction marker ZO-1 (Fig. 1*A* and *SI Appendix*, Fig. S1*A*). Furthermore, Homer1, the only Homer family member expressed in the kidney (58), displayed apical junctional staining in mouse renal epithelia (*SI Appendix*, Fig. S1 *B*–*D*), indicating that Homers are integral components of epithelial cell junctions in vivo.

Next, we investigated the molecular basis of the previously identified PATJ–Homer interaction (43). A conserved PxxF motif was identified in the linker between the PDZ5 and PDZ6 domains of PATJ (Fig. 1*B* and *SI Appendix*, Fig. S2*A*). Coimmunoprecipitation experiments in 293 T cells revealed that a PATJ fragment containing PDZ5–6 was sufficient to interact with endogenous Homers and that mutation of the PKGF motif abolished the interaction (Fig. 1*C*). Disruption of this motif in the PDZ1-8 fragment (Fig. 1*D*) and in full-length PATJ (*SI Appendix*, Fig. S2*B*) likewise eliminated Homer binding. Mutation of the ligand-binding pocket in the Homer1 EVH1 domain also disrupted the association with PATJ, as expected (*SI Appendix*, Fig. S2*C*) (59). Moreover, PATJ cofractionated with Homers in sucrose gradients and markedly increased the size or density of Homer assemblies, indicating that PATJ promotes Homer oligomerization (*SI Appendix*, Fig. S2*D*). To determine whether PATJ is required for the recruitment of Homers to the apical–lateral border, we analyzed the distribution of Homers in PATJ knockout (KO) MDCK cells (60). Loss of PATJ resulted in a marked displacement of all Homer proteins from apical cell junctions, whereas total Homer protein levels remained unchanged (Fig. 1*E* and *SI Appendix*, Fig. S2*E*). Together, these data demonstrate that PATJ recruits Homers to apical cell junctions via a direct EVH1–PxxF motif interaction.

### Homers and PATJ Coordinate YAP/TEAD and Wnt Signaling in Polarized Epithelial Cells.

To address the functions of Homers, we generated single and triple Homer KO MDCK cells using CRISPR/Cas9 (*SI Appendix*, Fig. S3 *A*–*F*). The localization of PATJ, Pals1, and several junctional proteins was not affected in Homer triple KO (TKO) cells, while cortical actin levels were slightly increased (*SI Appendix*, Fig. S3 *G* and *H*). Moreover, Homer TKO cells showed no defect in cell proliferation (*SI Appendix*, Fig. S3*I*) but were impaired in collective cell migration, as determined by scratch wound assays in confluent MDCK cell monolayers (*SI Appendix*, Fig. S3*J*).

To explore a function of Homers in signaling, we analyzed the transcriptome of Homer TKO cells using RNA sequencing (*SI Appendix*, Fig. S4*A* and Dataset S1). Interestingly, genes involved in cancer signaling were significantly deregulated in Homer TKO cells (*SI Appendix*, Fig. S4 *B*–*D*). Closer inspection of the differentially expressed genes (DEGs) showed that the canonical YAP/TEAD target genes CTGF, ANKRD1 and GADD45A (61) as well as additional potential YAP targets including FOLR2 (62), COL12A1 (63, 64), and HOXA2 (65) were downregulated in Homer TKO cells (*SI Appendix*, Fig. S4*E*). In addition, the expression of several genes involved in Wnt signaling was reduced in Homer TKO cells. This includes the Wnt receptor FZD4, the Wnt target genes HAS2 (66–69), ARHGAP24 (70), and POU3F3 (71), and GAS1 (72), DACH1 (73–75), PLEKHA4 (76), and GNA14 (77) (*SI Appendix*, Fig. S4*E*).

**Fig. 2.**
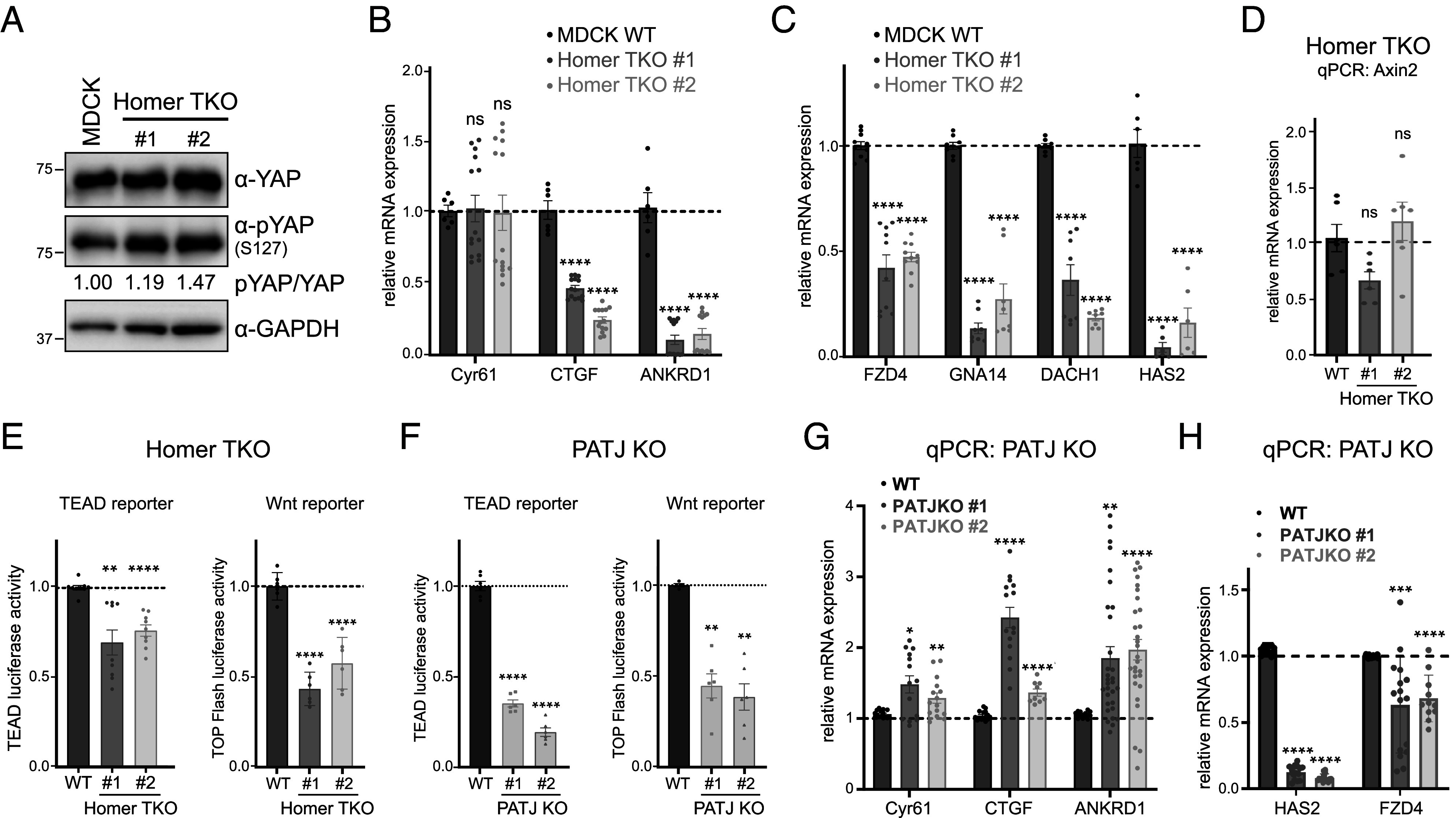
Homers and PATJ functionally interact to regulate YAP/TEAD and Wnt pathway activity (*A*) WB analysis of WT MDCK and Homer TKO cell lysates probed for YAP and pYAP (S127). The pYAP/YAP ratio was quantified by densitometry (n = 3). (*B* and *C*) qPCR analysis of canonical YAP target genes (*B*) and Wnt-associated genes (*C*) in Homer TKO cells (n = 4). (*D*) qPCR analysis of Axin2 expression in Homer TKO cells (n = 4). (*E* and *F*) TEAD and TOPFlash luciferase reporter assays in Homer TKO (*E*) and PATJ KO (*F*) cells (n = 3). (*G* and *H*) qPCR analysis of canonical YAP target genes (*G*) and Wnt-associated genes (*H*) in PATJ KO cells (n = 6). Luciferase assay data were analyzed using a paired Student’s *t* test. qPCR data were analyzed using Two-Way ANOVA. Data are presented as mean ± SEM. *P ≤ 0.05, **P ≤ 0.01, ***P ≤ 0.001, ****P ≤ 0.0001.

To verify the transcriptional changes observed by RNA sequencing, we performed qPCRs and analyzed Hippo/YAP and Wnt/β-catenin pathway activity using TEAD (8xGTIIC) and TCF/LEF (TOPFlash) luciferase reporter assays, respectively. As expected, CTGF and ANKRD1 expression was significantly reduced in Homer TKO cells (Fig. 2*B*), accompanied by a 1.2-1.5-fold increase in YAP S127 phosphorylation, indicating reduced YAP activation (Fig. 2*A*). FZD4, GNA14, DACH1, and HAS2 mRNAs were also downregulated in Homer TKO cells (Fig. 2*C*), whereas the expression of the canonical Wnt target gene AXIN2 was unchanged (Fig. 2*D*). Consistent with a role of Homers and PATJ in YAP and Wnt signaling, both TEAD and TCF/LEF reporter activities were markedly reduced in Homer TKO and PATJ KO cells (Fig. 2 *E* and *F*). Furthermore, loss of PATJ increased YAP target gene transcription (Fig. 2*G*) and decreased the mRNA levels of HAS2 and FZD4 (Fig. 2*H*). We concluded that Homers and PATJ functionally interact to control YAP target gene expression and Wnt pathway activity, possibly via TEAD-dependent and -independent mechanisms.

### Homers Promote YAP and Wnt Pathway Activity in 293 T and HCT116 Cells.

To understand whether Homers function synergistically or in a unique fashion, we examined single Homer KO cells. Whereas loss of *Homer3* reduced YAP/TEAD signaling and increased YAP S127 phosphorylation, inactivation of *Homer1* or *Homer2* unexpectedly enhanced YAP activation (*SI Appendix*, Fig. S4 *F*–*H*). Consistent with this, overexpression of Homer3—but not Homer1—stimulated both TEAD and TOPFlash reporter activity (*SI Appendix*, Fig. S4 *I*–*K*), indicating that Homer3 acts as the dominant pro-YAP/Wnt pathway regulator in MDCK cells.

We next analyzed the functions of Homers in cells lacking polarity cues. In contrast to polarized MDCK cells, siRNA-mediated silencing of individual or all three Homers in 293 T cells uniformly reduced YAP target gene expression and TOPFlash reporter activity, whereas overexpression of Homers was associated with elevated TEAD and TOPFlash reporter readouts (*SI Appendix*, Fig. S5 *A*–*E*). Notably, TEAD activation was independent of Homer-mediated actin bundling (47), as WT Homer3 and an actin binding-deficient mutant stimulated the reporter to a similar extent (*SI Appendix*, Fig. S5*F*). Together, these results indicated that Homers promote YAP and canonical Wnt signaling in polarized and nonpolarized epithelial cells, and that their paralogue-specific functions are cell-type or context-dependent.

Given the prominent role of YAP–Wnt pathway crosstalk in colorectal cancer (CRC) (22, 23, 25, 78), we next assessed Homer function in HCT116 cells. HCT116 cells expressed Homer1 and Homer3, but not Homer2 (*SI Appendix*, Fig. S5*G*). siRNA-mediated Homer depletion significantly reduced YAP/TEAD and TOPFlash reporter activity without markedly altering the pYAP/YAP ratio or total β-catenin levels (*SI Appendix*, Fig. S5 *H*–*J*). Importantly, the expression of canonical YAP and Wnt target genes was significantly decreased in cells treated with Homer siRNA (*SI Appendix*, Fig. S5 *K* and *L*). Moreover, and in agreement with our MDCK cell data, Homer knockdown did not affect cell proliferation (*SI Appendix*, Fig. S5*M*), but markedly impaired wound closure in scratch wound assays (*SI Appendix*, Fig. S5*N*). Together, these findings identify Homers as positive regulators of YAP and Wnt signaling in CRC cells.

### Homers Control YAP/TEAD Signaling Via the NDR Kinase Scaffolding Protein FRYL.

To identify proteins that could link Homers to the YAP and Wnt signaling pathways, we performed immunoprecipitation-mass spectrometry (IP–MS) of GFP tagged Homer3 stably expressed in MDCK cells. As expected, Homer1, Homer2, and PATJ were present in Homer3 IPs. We further identified the Furry-like protein (FRYL) as a Homer3 interacting protein (*SI Appendix*, Fig. S6 *A* and *B*). Furry, the FRYL paralogue, is a conserved scaffolding protein of Nuclear Dbf2-related (NDR1/2) kinases. Both Furry and NDR1/2 have been shown to regulate YAP (4, 5) and are critical for vertebrate (79) and invertebrate (80–84) development. Consistent with a previous report (85), FRYL and MOB1a specifically coimmunoprecipitated with GFP-NDR1 in 293 T cells (*SI Appendix*, Fig. S6*C*). In addition, overexpression of FRYL increased NDR Thr442/Thr444 phosphorylation (a readout for NDR kinase activity) but did not affect the phosphorylation of LATS1/2 (Thr1029) (*SI Appendix*, Fig. S6*D*). This establishes FRYL as a specific NDR1 scaffolding protein.

**Fig. 3.**
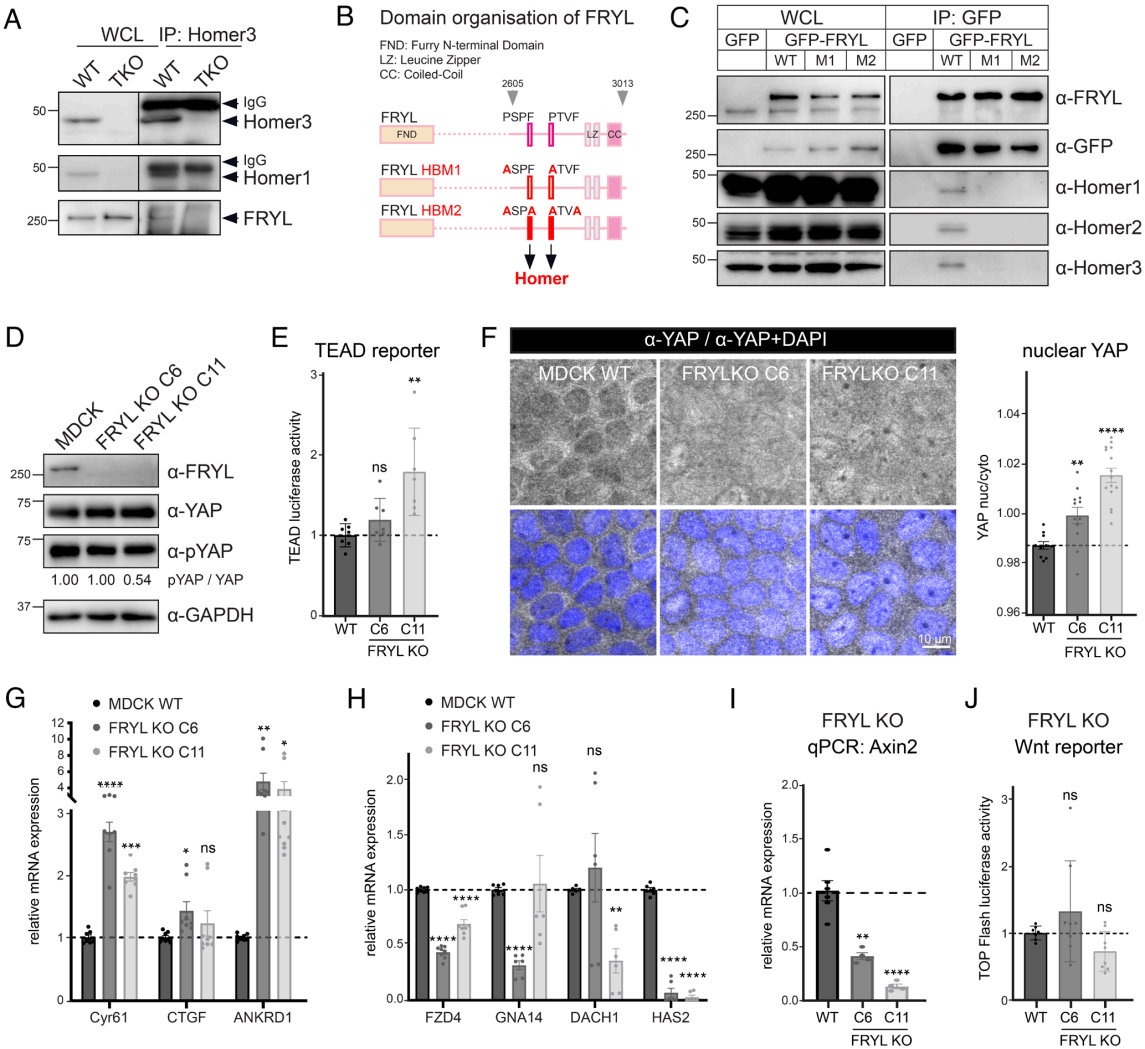
The NDR kinase scaffolding protein FRYL interacts with Homers and suppresses YAP/TEAD signaling (*A*) IP of the endogenous Homer/FRYL complex from MDCK cell lysates using anti-Homer3 antibodies. Lysates from Homer TKO cells were used as a control. (*B*) Domain organization of FRYL. The two Homer binding sites are depicted. The Homer binding mutants (HBM) M1 and M2 exhibit distinct mutations in the two PxxF motifs. (*C*) IP of WT GFP-FRYL and the two FRYL HBMs M1 and M2 from 293 T cell lysates. (*D*) WB analysis of MDCK WT and FRYL KO cell lysates probed for FRYL, YAP, and p-YAP (S127). The p-YAP/YAP ratio was quantified by densitometry (n = 3). (*E*) TEAD luciferase activity in FRYL KO cells normalized to MDCK WT cells (n = 3). (*F*) FRYL KO and MDCK WT cells were cultured on Transwell filters, fixed, and stained for total YAP. Nuclear and cytoplasmic YAP intensities were quantified using DAPI staining as a mask to define the nucleus. Quantification was performed on 10 images per condition, analyzing more than 80 cells per image (n = ~800 cells). Data are presented as mean ± SEM. **P ≤ 0.01, ****P ≤ 0.0001 (Student’s *t* test). (*G* and *H*) qPCR analysis of YAP target genes (*G*) and Wnt-associated genes (*H*) in FRYL KO cells (n = 3). (*I*) qPCR analysis of Axin2 mRNA levels in FRYL KO cells (n = 3). (*J*) TOPFlash luciferase activity in FRYL KO cells normalized to MDCK WT cells (n = 3). All Luciferase assays were statistically analyzed using a paired Student’s *t* test. All qPCR data were analyzed using Two-Way ANOVA Tukey multiple comparison test. Data are presented as mean ± SEM. *P ≤ 0.05; **P ≤ 0.01; ***P ≤ 0.001; ****P ≤ 0.0001.

To confirm that Homers interact with FRYL, we immunoprecipitated endogenous Homer3 from MDCK cell lysates. As expected, Homer1 and FRYL were present in Homer3 IPs, indicating that Homer oligomers form a complex with FRYL in polarized epithelia (Fig. 3*A*). We noted that the C-terminus of FRYL harbors two highly conserved PxxF motifs that could potentially function as Homer binding sites (Fig. 3*B* and *SI Appendix*, Fig. S6 *E*–*G*). Indeed, WT GFP-FRYL efficiently interacted with all three Homers in IPs, whereas no interactions were observed with two independent Homer binding mutants (HBMs) in which the two PxxF motifs were mutated (Fig. 3*C*). Additional experiments showed that the FRYL C-terminus (aa 2605-3013) was sufficient for Homer binding and that mutations in the first (PSPF) or the second (PTVF) Homer binding site completely disrupted the interaction, indicative of a cooperative binding mechanism (*SI Appendix*, Fig. S6 *H* and *I*). Furthermore, Homers did not co-IP with Furry, confirming the specificity of the interaction. We concluded that FRYL interacts directly and specifically with Homers via two PxxF motifs.

To explore a potential role of FRYL in YAP signaling, we produced *FRYL* MDCK KO cells. Interestingly, loss of FRYL reduced YAP S127 phosphorylation and increased both TEAD reporter activity and YAP nuclear translocation in at least one out of the two KO clones examined (Fig. 3 *D*–*F*). Moreover, genetic inactivation of *FRYL* in MDCK cells as well as siRNA-mediated silencing of FRYL in 293 T or HCT116 cells significantly increased YAP target gene transcription (Fig. 3*G* and *SI Appendix*, Fig. S7), demonstrating that FRYL suppresses YAP/TEAD signaling. By contrast, the expression of the Wnt-associated genes FZD4, GNA14, DACH1, and HAS2 was reduced in *FRYL* KO cells (Fig. 3*H*). Axin2 mRNA levels were also decreased but Wnt reporter activity was unexpectedly unchanged (Fig. 3 *I* and *J*). This indicates that Homers and FRYL function antagonistically in regulating the transcription of canonical YAP/TEAD target genes but synergize to control Wnt-associated genes.

### Homers Form Biomolecular Condensates in 293 T and HCT116 Cells.

**Fig. 4.**
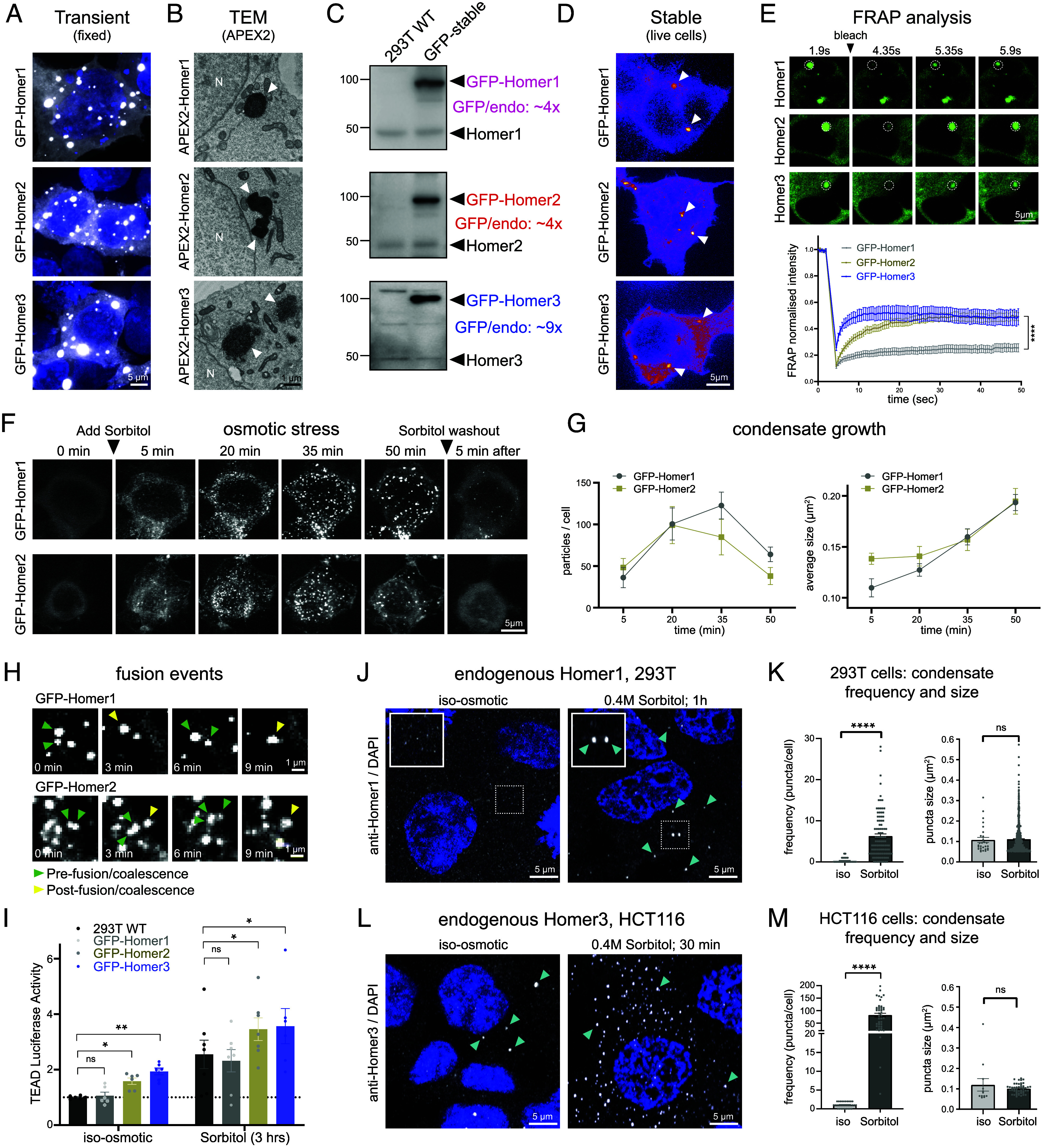
Homers form cytoplasmic biomolecular condensates at endogenous levels (*A*) Confocal micrographs of fixed 293 T cells transiently transfected with GFP-Homer1, 2, or 3. (*B*) Representative transmission electron micrographs of 293 T cells transfected with APEX2-EGFP tagged Homer1, Homer2, or Homer3. EM contrast of Homer condensates was enhanced through APEX2 labeling. Note the distinct ultrastructure of Homer condensates. N = nucleus. (*C*) Western blot analysis of 293 T cells stably transfected with GFP-Homer1, 2, or 3. The degree of overexpression was determined by densitometry as GFP-Homer over endogenous Homer signal (n = 3). (*D*) Live cell spinning disk microscopy of 293 T cells stably transfected with GFP-Homer1, 2, or 3. Note the presence of small cytoplasmic puncta. (*E*) FRAP analysis of GFP-Homer1, 2, and 3 puncta in stably transfected 293 T cells (n = 20). (*F*) Stable GFP-Homer cells were imaged live by spinning disk microscopy before and after the addition of 0.4 M sorbitol for 1 h. (*G*) Quantification of particle size (area) and frequency before and after induction of hyperosmotic shock based on data shown in (*F*) (n = 10). (*H*) Fusion events of GFP-Homer1 and GFP-Homer2 puncta in stable 293 T cells. (*I*) TEAD luciferase assay in WT 293 T cells and stable GFP-Homer cell lines grown in isosmotic medium or in medium containing 0.4 M sorbitol for 3 h. Note that overexpression of GFP-Homer2 or GFP-Homer3 enhances TEAD activity under isosmotic and hyperosmotic conditions. (*J*) Airyscan microscopy of fixed 293 T cells grown in isosmotic medium or in medium containing 0.4 M sorbitol for 1 h stained with Homer1 antibodies. (*K*) Quantification of Homer1 puncta size and frequency based on data shown in (*J*). (*L*) Airyscan microscopy of fixed HCT116 cells grown in isosmotic medium or in medium containing 0.4 M sorbitol for 30 min stained with Homer3 antibodies. (*M*) Quantification of Homer3 puncta size and frequency based on data shown in (*L*). Data in *K* and *M* are presented as mean ± SEM. ****P ≤ 0.0001 (Student’s *t* test; n = 33 to 50 cells each).

To understand how Homers regulate YAP and Wnt signaling, we analyzed their localization and dynamics in 293 T cells. Interestingly, transient overexpression of GFP-tagged Homers in 293 T cells produced large cytoplasmic puncta (Fig. 4*A* and *SI Appendix*, Fig. S8*A*). Live cell imaging revealed that these structures grow through fusion and coalescence (*SI Appendix*, Fig. S8 *C* and *D*). The puncta were devoid of a surrounding membrane and displayed considerable ultrastructural heterogeneity, as determined by transmission electron microscopy (Fig. 4*B* and *SI Appendix*, Fig. S8 *E* and *F*). In addition, Fluorescence Recovery After Photobleaching (FRAP) showed that Homers are largely immobile within these assemblies, with only 13 to 26% of Homer fluorescence recovery within 1 min after photobleaching (*SI Appendix*, Fig. S8*G*).

To determine if Homers phase separate at or close to physiological expression levels, we generated stable 293 T cell lines expressing GFP-Homer1 and GFP-Homer2 at ~fourfold above endogenous levels and GFP-Homer3 at ~ninefold over baseline (Fig. 4*C*). All GFP-Homer proteins produced cytoplasmic puncta in live cells, though at markedly lower frequency than after transient overexpression (Fig. 4*D*). FRAP revealed substantially greater mobility for GFP-Homer2 and GFP-Homer3 (~50% recovery within 20 s upon photobleaching), whereas GFP-Homer1 remained largely immobile (Fig. 4*E*). This indicates that Homers assemble condensates with variable ultrastructural and dynamic properties.

Hyperosmotic stress (or macromolecular crowding) has been shown to promote the phase separation of Hippo signaling molecules (11, 14) and to enhance YAP/TEAD activity (12, 86). Interestingly, addition of 0.4 M sorbitol to live 293 T cells stably expressing GFP-Homers triggered a rapid and fully reversible increase in the number of cytoplasmic puncta (Fig. 4*F* and Movie S1). Over time, the number of puncta decreased while their average size increased, consistent with coarsening (Fig. 4*G*). Fusion events between individual puncta were frequently observed (Fig. 4*H* and Movie S2), supporting their dynamic and liquid-like behavior.

To test whether Homer condensates promote YAP/TEAD signaling, we performed TEAD luciferase assays in WT and stable GFP-Homer 293 T cells grown under isosmotic or hyperosmotic conditions. Osmotic stress enhanced TEAD activity in WT 293 T cells, as expected (12, 86) (Fig. 4*I*). In line with our data from transient overexpression (*SI Appendix*, Fig. S5*D*), stable overexpression of GFP-Homer2 or GFP-Homer3 (but not GFP-Homer1) significantly increased TEAD activity under basal conditions, and a further increase was observed in response in sorbitol-treated cells (Fig. 4*I*). We concluded that Homer proteins promote YAP/TEAD signaling through phase separation.

Next, we examined endogenous Homer1 in fixed 293 T cells using Airyscan microscopy (which offers a lateral resolution of ~120 nm). Under isosmotic conditions, Homer1 immunostaining appeared predominantly diffuse and cytoplasmic puncta were rarely detected. Intriguingly, osmotic stress markedly increased the number of Homer1 puncta (Fig. 4*J*). These structures were ~350 nm in diameter (average area ~0.1 µm^2^) (Fig. 4*K*) and therefore similar in size to the puncta observed in sorbitol-treated GFP-Homer–expressing cells (diameter ~500 nm; average area ~0.2 µm^2^) (Fig. 4*G*). Furthermore, immunostaining of Homer3 in HCT116 cells [which express substantially higher levels of Homer3 than 293 T cells (*SI Appendix*, Fig. S5*G*)] revealed distinct cytoplasmic puncta even under isosmotic conditions (Fig. 4*L*), and the frequency of these puncta increased dramatically in response to osmotic stress (Fig. 4*M*). Taken together, these data demonstrate that Homer proteins phase separate at endogenous expression levels.

### FRYL and PATJ Differentially Regulate Homer Phase Separation.

Given that YAP phase separates with Hippo pathway regulators in the nucleus and the cytoplasm (7–14), we wondered whether Homer condensates sequestered YAP. We found that YAP-GFP colocalized with Homers when both proteins were co-overexpressed (*SI Appendix*, Fig. S9 *A* and *B*). However, endogenous YAP was not detected in Homer condensates induced by osmotic stress (*SI Appendix*, Fig. S9 *C* and *D*) and YAP-GFP condensates induced by osmotic stress did not colocalize with endogenous Homers (*SI Appendix*, Fig. S9*E*). This indicates that, under physiological conditions, YAP is largely excluded from Homer condensates.

**Fig. 5.**
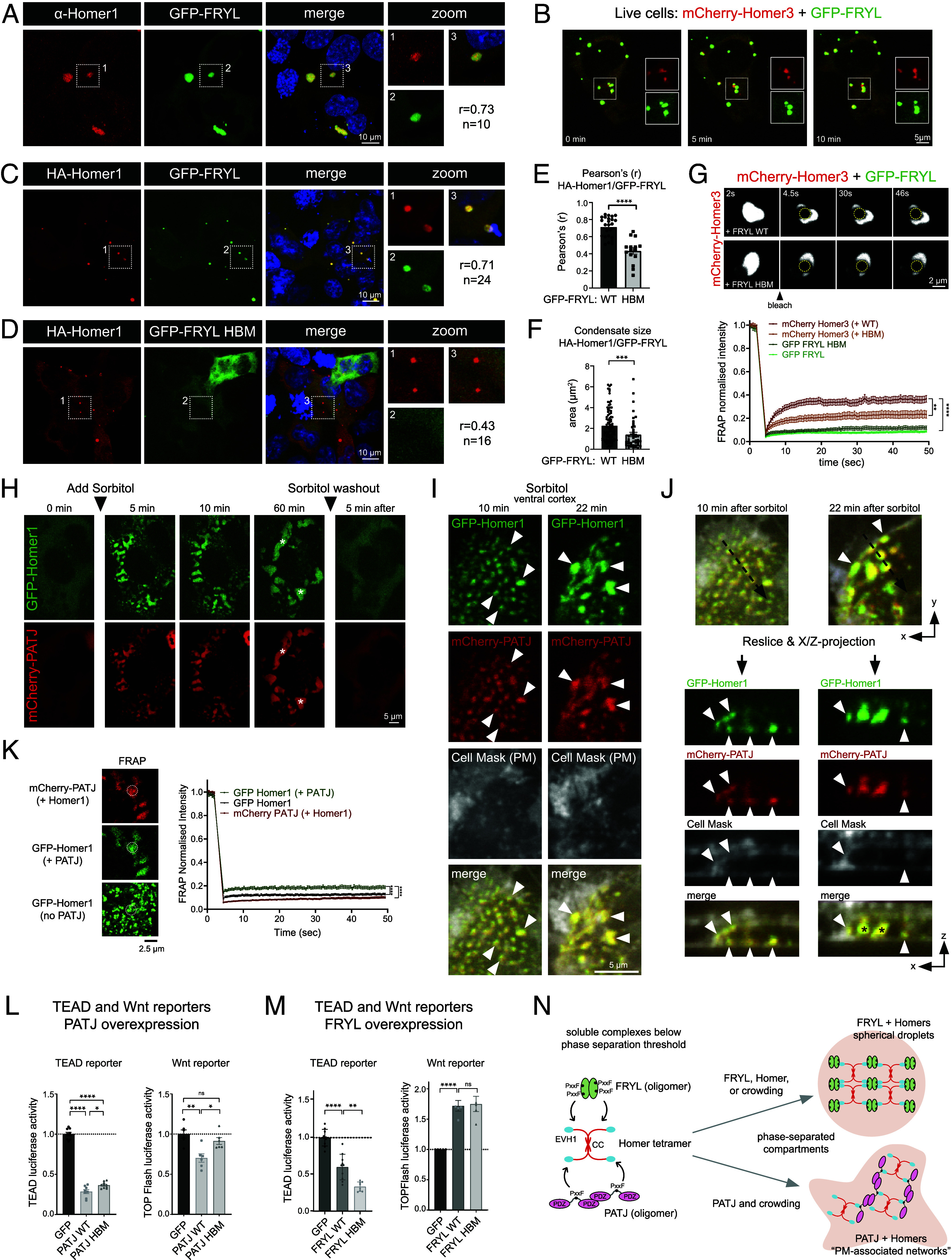
FRYL and PATJ differentially regulate Homer phase separation (*A*) 293 T cells transiently transfected with GFP-FRYL were fixed and stained with Homer1 antibodies. (*B*) Live cell microscopy of 293 T cells cotransfected with mCherry-Homer3 and GFP-FRYL. (*C* and *D*) 293 T cells were cotransfected with HA-Homer1 and GFP-FRYL (*C*) or the GFP-FRYL HBM M2 (*D*). (*E*) Pearson’s correlation (r) analysis of HA-Homer1 and GFP-FRYL vs. HA-Homer1 and GFP-FRYL HBM M2. (*F*) Size measurements of HA-Homer1 condensates in cells cotransfected with GFP-FRYL or GFP-FRYL HBM M2. (*G*) FRAP analysis of mCherry-Homer3 in 293 T cells cotransfected with either GFP-FRYL or GFP-FRYL HBM M2 (n = 17). (*H*) Live cell confocal microscopy of stable GFP-Homer1 293 T cells transfected with mCherry-PATJ before and after sorbitol addition and after sorbitol washout. (*I*) Live cell confocal microscopy of stable GFP-Homer1 293 T cells transfected with mCherry-PATJ stained with the plasma membrane dye CellMask. Confocal z-stacks were acquired 10 min after sorbitol addition, using a time interval of 3 min and a z-interval of 200 nm. (*J*) Orthogonal X/Z projections of the data shown in (*I*). Arrowheads highlight membrane-associated condensates, asterisks indicate cytoplasmic condensates. (*K*) FRAP analysis of stable GFP-Homer1 293 T cells transfected with mCherry-PATJ. All FRAP measurements were acquired 5 to 30 min after sorbitol addition (n = 34). (*L*) TEAD and TOPFlash reporter assays in 293 T cells transfected with GFP, WT GFP-PATJ PDZ1-8, or GFP-PATJ PDZ1-8 HBM (n = 4). (*M*) TEAD and TOPFlash reporter assays of 293 T cells transfected with GFP, WT GFP-FRYL, or GFP-FRYL HBM M2 (n = 5). FRAP data in (*G*) and (*K*) is presented as mean ± SEM. Statistical analysis was performed using two-way ANOVA. Luciferase assay data were analyzed using a paired Student’s *t* test. *P ≤ 0.05, **P ≤ 0.01, ***P ≤ 0.001, ****P ≤ 0.0001. (*N*) Model of how osmotic stress and changes in Homer, PATJ, and FRYL expression modulate Homer phase separation. FRYL is depicted as a hypothetical dimer but could equally enhance valency as a monomer (due to its two Homer binding sites). PATJ is depicted as PDZ5-6 fragment for simplicity and depicted as a putative oligomer (see discussion).

Next, we addressed the functions of FRYL and PATJ in Homer phase separation. Interestingly, transient overexpression of GFP-FRYL, but not the FRYL HBM, resulted in the spontaneous formation of cytoplasmic droplets (*SI Appendix*, Fig. S8*B*). Endogenous Homer1 was efficiently recruited into these structures (Fig. 5*A*), indicating that FRYL promotes Homer phase separation even in the absence of osmotic stress. GFP-FRYL also colocalized with mCherry-Homer3 in live cells (Fig. 5*B* and Movie S3) and with HA-tagged Homer1 in fixed cells (Fig. 5*C*), and colocalization was significantly reduced when the two Homer binding sites in FRYL were mutated (Fig. 5 *D* and *E*). We further found that Homer1 condensates were significantly larger in cells cotransfected with wild-type FRYL compared to cells cotransfected with the FRYL HBM (Fig. 5*F*). Moreover, FRAP demonstrated that WT (but not mutant) FRYL significantly increased the mobile fraction of Homer3 from ~25 to ~36% (Fig. 5*G*). FRYL itself exhibited minimal fluorescence recovery (~10% mobile fraction), indicating a tight association with the Homer scaffold. We concluded that FRYL promotes Homer phase separation in a PxxF motif-dependent manner.

In contrast to FRYL, overexpressed mCherry-PATJ did not promote Homer condensation under isotonic conditions. Upon osmotic stress, however, the two proteins rapidly partitioned into large networks morphologically distinct from the cytoplasmic droplets induced by FRYL (Fig. 5*H*, *SI Appendix*, Fig. S10*A*, and Movie S4). Sorbitol washout rapidly dissolved these structures, demonstrating a fully reversible assembly mechanism (Fig. 5*H*). Live-cell imaging revealed that at least a subset of PATJ-Homer condensates emerged from the coalescence of small nucleation sites at or near the plasma membrane (Fig. 5 *I* and *J* and *SI Appendix*, Fig. S11 *A*–*C*). Both Homer1 and PATJ were largely immobile within these structures (<20% mobile fraction), with PATJ slightly increasing the mobility of Homer1 compared to that observed in cytoplasmic droplets (Fig. 5*K* and *SI Appendix*, Fig. S11*D*). We then co-overexpressed PATJ PDZ1-8 and mCherry-Homer3, which resulted in the formation of cytoplasmic droplet-like condensates (*SI Appendix*, Fig. S10*B* and Movie S5). As expected, mutation of the Homer-binding site in PATJ PDZ1-8 significantly reduced its colocalization with Homer3 (*SI Appendix*, Fig. S10*C*). Moreover, PATJ PDZ1-8 was more mobile in droplets (~40% mobile fraction) than in PM-associated condensates and did not affect the mobility of Homer3 (*SI Appendix*, Fig. S10*D*). Together, these findings demonstrate that FRYL and PATJ differentially regulate Homer phase separation, producing condensates with distinct morphologies and dynamic properties.

Next, we examined whether the functions of PATJ and FRYL in YAP/TEAD and Wnt signaling depend on their direct interactions with Homers. Interestingly, overexpression of PATJ PDZ1-8 in 293 T cells significantly reduced both TEAD and TOPFlash activity, and mutation of the Homer binding site partially (TEAD) or completely (TOPFlash) reversed this effect (Fig. 5*L*). Overexpression of FRYL also reduced TEAD reporter activity, and its inhibitory effect was significantly enhanced when the Homer binding site in FRYL was inactivated (Fig. 5*M*). In contrast, TOPFlash reporter activity was increased upon overexpression of FRYL, independently of its association with Homers (Fig. 5*M*). Collectively, our data demonstrate that PATJ and FRYL modulate YAP and Wnt pathway output via Homer-dependent and -independent mechanisms.

## Discussion

Biomolecular condensation has emerged as a key regulatory mechanism of Hippo/YAP and Wnt/β-catenin signaling (8, 87–90). Here, we identify Homer proteins as phase separating scaffolds that promote YAP and Wnt signaling in epithelial and cancer cells. Despite their high sequence similarity, Homer1, Homer2, and Homer3 condensates varied in ultrastructure and exhibited different FRAP kinetics. Their effects on YAP/TEAD signaling were similarly heterogeneous and cell-type dependent. This suggests that the three Homer paralogues are not functionally redundant and that relative abundance, and perhaps subtle differences in ligand binding or oligomerization behavior, modulate the condensates’ organization and signaling output (91, 92). We also demonstrate that PATJ and FRYL differentially regulate Homer condensate assembly (Fig. 5*N*). FRYL promoted the formation of cytoplasmic Homer droplets even under isotonic conditions, indicating that it acts as a multivalent coscaffold that enhances connectivity within the Homer tetramer network. By contrast, PATJ did not phase separate under isotonic conditions but rapidly coalesced with Homers into plasma membrane-associated assemblies following osmotic stress. These observations suggest that PATJ promotes Homer condensation specifically at the cell cortex, which likely involves PATJ clustering and oligomerization via associated binding partners, PDZ–PDZ interactions (93–95), or PDZ-mediated lipid binding (96, 97). Whether FRYL and PATJ are sufficient to drive Homer condensation or act together with additional cellular factors remains to be explored. In fact, we anticipate that other scaffolds and clients, recruited via FRYL and PATJ or sequestered via independent interactions, further shape the assembly and signaling output of Homer condensates (98–100). Given that Hippo kinases and several upstream Hippo pathway regulators control YAP activity through phase-separated assemblies (9, 11, 14), an important question for future studies is whether Homer condensates are related to these structures or instead represent a distinct class of biomolecular condensate.

Functionally, our data indicate that Homers, PATJ, and FRYL regulate YAP/TEAD signaling through interconnected inhibitory interactions. We propose that Homer condensates sequester and suppress the FRYL–NDR complex, thereby limiting NDR-dependent YAP phosphorylation and promoting YAP/TEAD-driven transcription. Although a direct role for NDR downstream of Homers remains to be established, this model is consistent with studies demonstrating that NDR phosphorylates and inhibits YAP in both the mouse intestine and cultured cells (4, 5). PATJ, in contrast, antagonizes the pro-YAP activity of Homers, potentially by recruiting Homers to the plasma membrane. Since PATJ depletion in MDCK cells enhanced endogenous YAP target gene expression while simultaneously reducing TEAD reporter activity, PATJ is likely to influence Hippo pathway output through additional mechanisms. For instance, PATJ may act through TAZ, alternative transcriptional regulators such as TCF/LEF, AP-1, and SMAD2/3 (40, 101–103), or via the Hippo pathway regulators AMOT and KIBRA (104–107). Altogether, our data are consistent with the established role of the Crumbs complex in cell density-dependent Hippo pathway regulation (32, 39, 40) and raise the possibility that PATJ links tight junction formation and polarity establishment to YAP inhibition by promoting ZO-1 and Homer condensation at the apical–lateral border (108–110).

**Fig. 6.**
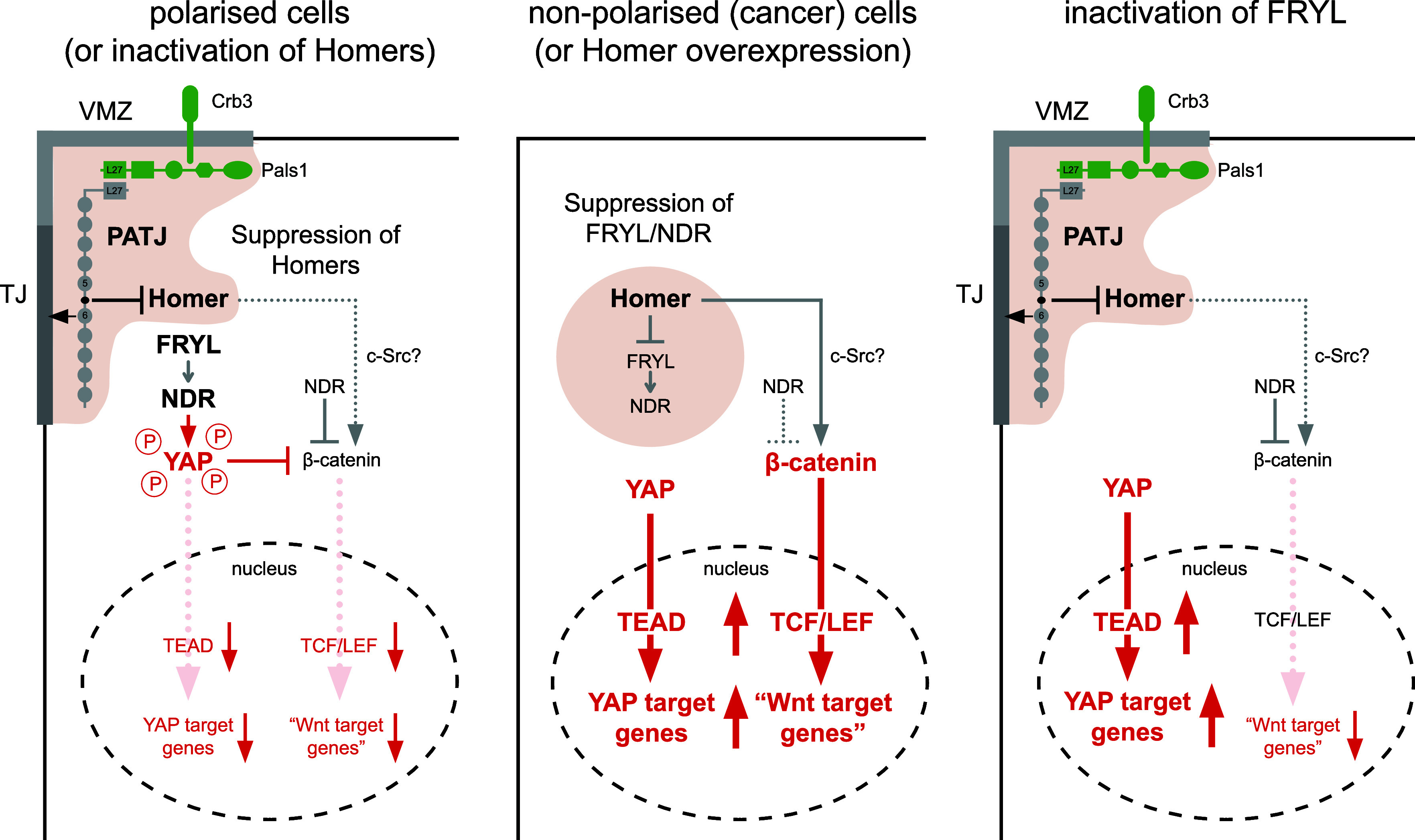
Model of Homer-mediated YAP–Wnt signaling crosstalk Hypothetical mechanism of how PATJ, Homers, and FRYL control YAP–Wnt signaling crosstalk. The model predicts that Homers suppress the FRYL/NDR complex in cytoplasmic condensates and that PATJ recruits Homers to the membrane to shut down Homer-mediated YAP activation through membrane recruitment (see discussion).

Our data further indicate that the PATJ–Homer–FRYL axis links YAP regulation to canonical Wnt/β-catenin signaling. Importantly, downregulation of Homers in HCT116 cells suppressed both YAP/TEAD and Wnt pathway activity as well as cell migration. This suggests a tumor-promoting role for Homers and supports a model in which YAP activity and Wnt signaling are positively coupled in colorectal cancer cells (22, 23, 25, 78). While Homers and FRYL acted antagonistically on classical YAP target genes, both enhanced the expression of several pro-oncogenic, Wnt-associated genes, including FZD4, GNA14, DACH1, and HAS2 (66–69, 74, 75, 77, 111–113). FRYL-mediated suppression of YAP was modulated by its interaction with Homers, but its Wnt-promoting activity was not. This suggests that Homers and FRYL converge to regulate YAP but influence β-catenin activity via additional, YAP-independent mechanisms (57, 114, 115). Collectively, we propose a model in which Homer condensates act as tunable signaling platforms that integrate polarity cues with Hippo and Wnt pathway activity (Fig. 6). In polarized epithelial cells, PATJ restrains Homer-driven YAP activation, thereby enhancing YAP phosphorylation and cytoplasmic retention, which in turn dampens Wnt signaling via YAP-β-catenin crosstalk (20, 21, 23). In cancer cells—where Homers are often upregulated and polarity is disrupted—this restraint may be lost, resulting in altered condensate properties that may shift signaling toward persistent YAP and Wnt activation. Homer condensates may therefore represent targets for modulating oncogenic YAP and β-catenin signaling in epithelial-derived cancers.

## Materials and Methods

### Confocal Microscopy and Live-Cell Imaging.

Fixed cell imaging of condensates was performed on a Zeiss LSM980 confocal microscope using a 63× oil immersion objective (Zeiss, NA 1.4) and Airyscan detection. The images were scanned at 940 × 940 pixels, unidirectionally with SR-8Y mode. Detection gain was within the range of 650 to 850. Confocal images were gain-corrected and analyzed in Image/Fiji (116). Live cell imaging was performed in phenol red–free DMEM supplemented with 10% FBS in an environmental control chamber maintaining cells at 37 °C and 5% CO2 using a CorrSight spinning disk microscope (Thermo Scientific) equipped with an Orca R2 CCD camera (Hamamatsu). Imaging was performed using 40× (EC Plan-Neofluar 40×/NA 1.3, Oil M27; Zeiss) or 63× (Plan-Apochromat 63×/NA 1.4, Oil M27; Zeiss) immersion objectives utilizing 405, 488, 561 nm, and 633 nm lasers for excitation and standard emission filters. Cells were imaged using minimal laser power and exposure times to prevent photobleaching and phototoxicity. Z-stacks were acquired using a 0.2 to 1 µm step size to visualize condensates throughout the cell volume. Time-lapse data were analyzed in Image/Fiji (116).

### Hyperosmotic Stress Induction.

For osmotic stress experiments, cells were allowed to reach 80% confluency prior to treatment. Hyperosmotic stress was induced by supplementing prewarmed culture medium with D-sorbitol (Sigma # S1876) to a final concentration of 0.4 M. Control cells were maintained in parallel under identical conditions without sorbitol treatment. To preserve sorbitol-induced condensates for fixed cell imaging, cells were gently washed once with PBS supplemented with 0.4 M sorbitol and then fixed with 4% PFA prepared in PBS containing 0.4 M sorbitol for 15 min at room temperature.

### Fluorescence Recovery After Photobleaching.

HEK293 T cells were seeded onto fibronectin-coated µ-slide 8-well glass bottom dishes (Ibidi # 80827-90) at a density of 1 × 10^5^ cells per well and transfected with a total of 0.26 µg of DNA using 1.3 µL of PEI as transfection reagent. FRAP experiments were conducted 24-h posttransfection in phenol red–free DMEM supplemented with 10% FBS. Live-cell imaging and FRAP were carried out using a 40× oil objective on Zeiss LSM 980 controlled by ZEN blue software. Fluorescence excitation was achieved using 587 nm for mCherry and 488 nm for GFP laser. A circular region of interest (ROI) with a diameter of 1.5 µm was selected within a condensate. Prebleach images were acquired at 0.5 s intervals for 2 s prior to photobleaching. Photobleaching was performed using 100% laser power for 2.45 s to bleach the fluorescence within the ROI. Postbleach recovery was monitored by acquiring images at 0.5 s intervals for up to 50 s, using minimal laser intensity to reduce phototoxicity and further bleaching. Fluorescence intensities were quantified using ZEN blue software by measuring the mean gray value within the bleached ROI and a reference ROI outside the bleached area to correct for overall photobleaching. Intensities were normalized to prebleach value and plotted as a function of time. Data were obtained from at least ten condensates per condition and plotted as mean ± SEM. Statistical analysis was performed using two-way ANOVA in GraphPad Prism.

### Quantifications and Statistical Analysis.

All statistical analyses were conducted using a two-tailed paired Student’s *t* test in GraphPad Prism, except for qPCR analysis, which was performed using a Two-way ANOVA with Tukey’s multiple comparison tests. FRAP statistical analysis was performed using Two-way ANOVA. Significance of data is represented as ns, not significant, *P ≤ 0.05, **P ≤ 0.01, ***P ≤ 0.001, ****P ≤ 0.0001. Error bars in all figures represent mean +/− SEM.

## Supplementary Material

Appendix 01 (PDF)

Dataset S01 (XLSX)

Movie S1. Formation of GFP-Homer1 condensates in response to osmotic stress.

Movie S2. Fusion of GFP-Homer1 condensates in sorbitol-treated cells.

Movie S3. GFP-FRYL and mCherry-Homer3 form droplet-like condensates in live cells.

Movie S4. mCherry-PATJ and GFP-Homer1 rapidly coalesce into interconnected networks upon osmotic stress induction.

Movie S5. GFP-PATJ PDZ1-8 and mCherry-Homer3 form droplet-like condensates in live cells.

## Data Availability

RNAseq, Microscopy, qPCR data have been deposited in DR-NTU (https://doi.org/10.21979/N9/BRU8EC) (117). All other data are included in the manuscript and/or supporting information.
