# Supplementary material for: Homer condensates orchestrate YAP–Wnt signaling crosstalk downstream of the Crumbs polarity complex
Source: Proc Natl Acad Sci U S A. 2026 Jul 15;123(29):e2523230123. doi: 10.1073/pnas.2523230123 (PMC13389695; doi:10.1073/pnas.2523230123)
Supplement: Supplementary file 1 — Appendix 01 (PDF) [file pnas.2523230123.sapp.pdf]

Supplemental Information

Dataset S1: RNA sequencing data of MDCK Homer triple KO cells compared to wild type cells. Sheet 1 shows all differentially expressed genes. Deregulated genes confirmed by qPCR are highlighted in yellow. Sheet 2 shows a heatmap of the top 100 downregulated and upregulated genes (related to Fig. S4A).

Supplemental Movies:
Movie S1: Formation of GFP-Homer1 condensates in response to osmotic stress
Movie S2: Fusion of GFP-Homer1 condensates in sorbitol-treated cells
Movie S3: GFP-FRYL and mCherry-Homer3 form droplet-like condensates in live cells
Movie S4: mCherry-PATJ and GFP-Homer1 rapidly coalesce into interconnected networks upon osmotic stress induction
Movie S5: GFP-PATJ PDZ1-8 and mCherry-Homer3 form droplet-like condensates in live cells

Supporting Information Text: Materials and Methods

Supplemental Tables:
Table S1: qPCR primers, plasmids, and oligonucleotides
Table S2: Materials and Resources. List of all antibodies, guideRNAs, siRNAs, cell lines, software, and recombinant DNA

Supplemental Figures S1-S11

Supporting Information Text

Materials and Methods

Cell culture

Madin-Darby Canine Kidney type II (MDCK-II), Human Embryonic Kidney 293T (HEK293T), and three human colorectal carcinoma cell lines (HCT116, DLD-1, and RKO) were used in this study. MDCK-II, HEK293T and HCT116 cells were grown in DMEM, high glucose, GlutaMAX™ pyruvate (Gibco #10569010). DLD-1 and RKO cells were grown in RPMI 1640 supplemented with L-Glutamine (Gibco #11875093). All media were supplemented with 10 % foetal bovine serum and 100 U/ml penicillin-streptomycin. All cells were grown at 37 ^{O}C and under 5% CO_2. Cell lines used were not authenticated and were negative to mycoplasma contamination. For some experiments MDCK cells were seeded onto polycarbonate or polyethylene 0.4 μm-pore Transwell® filter inserts at a seeding density of approximately 2.2×10^5 cells/cm^2. Cells were grown on the filter for 10-14 days to achieve full polarisation. Media was replenished every two days.

Bacterial strains

NEB10-beta competent *E. coli* (NEB, #C3019I) were used to transform plasmids for cloning. Bacteria were cultured in LB Agar (BD, Difco #244520) and LB Broth (BD, Difco #244620) at 37°C.

Generation of MDCK knockout cells

Stable MDCK cell lines expressing EGFP-APEX2 tagged Homers (1) and PATJ knockout MDCK cells (2) have been described previously. To generate Homer and FRYL knockout cells, MDCK cells were co-transfected with 0.5 μg of PX459 plasmid (encoding Cas9 and the single-guide RNA targeting the gene of interest) and 0.1 μg of pEGFP-C1. The pEGFP-C1 plasmid encoded a neomycin (G418/geneticin) resistance cassette, allowing for antibiotic selection. 24 hours after transfection, cells were placed under 200 μg/mL G418 selection for 14 days. Surviving cells were then sorted by FACS based on GFP expression and plated as single-cell clones in 96-well plates. Clonal populations were expanded and screened for knockout efficiency by Western blotting to confirm the absence of the target protein. Knockout clones were further validated by Sanger sequencing of the targeted locus and by immunofluorescence staining to confirm the loss of protein expression at the cellular level.

DNA constructs

Homers, PATJ, FRYL, and NDR1 cDNAs were PCR amplified from commercial vectors, utilizing primers specifically designed to enable seamless ligation into the pEGFP-C1, p3xHA-C1, or pEGFP-APEX2-C1 backbones (1). PATJ and NDR1 constructs were generated using Transfer PCR, using www.rf-cloning.org/ for primer design. Details of cDNA cloning and primers are listed in Table S1. Full-length FRYL was cloned into the pEGFP-C1 backbone using Gibson Assembly HiFi DNA Assembly Cloning Kit (NEB #E5520). Site-directed mutagenesis PCR was used to introduce specific mutations into the wild-type cDNAs. For PATJ, mutations P812A and F815A were introduced to inactivate the PxxF motif in the linker region between PDZ domains 5 and 6. Homer1 was mutated at position G89A, rendering the EVH1 domain inactive. The two PxxF motifs in FRYL were mutated to produce the mutants M1 (P2663A &

P2757A) and M2 (P2663A & F2666A / P2757A & F2760A). The specific cDNAs and primers used for these mutagenesis reactions are detailed in Table S1. CRISPOR (http://crispor.org) was used to design gRNA. Three gRNAs targeting early exons were selected for each gene of interest. The gRNAs were synthesized as pairs of short oligonucleotides with 5' phosphorylated ends. These oligonucleotide pairs were annealed in annealing buffer (1 mM Tris pH 8, 5 mM NaCl, 0.5 mM EDTA in Milli-Q water) by heating the mixture to 95°C, followed by a gradual cooling to 30°C for 1 hour. The annealed gRNAs were then ligated into pSpCas9(BB)-2A-Puro (PX459) (Addgene #62988) vector digested with *BbsI*-HF® (NEB). All gRNAs used in this study are listed in Table S2. All plasmids were transformed into NEB10β (NEB #C3019I) competent *E.coli*. All plasmids were screened by restriction enzyme digest or PCR and verified by DNA sequencing.

Immunofluorescence

HEK293T cells were seeded onto fibronectin-coated glass coverslips to promote robust adhesion. MDCK cells were cultured either on glass coverslips or on Transwell® filters, depending on the experimental setup. Cells were washed three times with PBS++ (PBS supplemented with Ca^{2+} and Mg^{2+}) and fixed with either 1% or 4% paraformaldehyde (PFA), depending on the primary antibody used, in PBS++ for 10-20 minutes at room temperature, followed by three additional washes with PBS++. Cells were then permeabilised with 0.5% Triton X-100 in PBS for 10-15 minutes, washed three times in PBS, and subsequently blocked in 10% FBS in PBS for 1 hour at room temperature. For Transwell-grown monolayers, the membrane filters were carefully excised using sterile razor blades and handled with the cell-facing side up throughout all staining steps. All incubation steps, including washes, were performed in a humidified chamber to prevent drying.

Samples were incubated with primary antibodies diluted in antibody incubation buffer (0.2 µm-filtered PBS containing 0.1% BSA and 0.01% Tween-20) for 1–3 hours at room temperature. After three PBS washes, samples were incubated with fluorophore-conjugated secondary antibodies diluted in the same buffer for 1 hour at room temperature, protected from light. Following secondary antibody incubation, samples were stained with 20 ng/mL DAPI in PBS to label nuclei and washed thoroughly. Glass coverslips were mounted onto microscope slides using mounting medium (Vectashield, Vector Laboratories H-1000). For Transwell® samples, the membranes were placed cell side up, overlaid with mounting medium, and covered with #1.5 (12 mm) glass coverslips (VWR). Coverslips were sealed using transparent nail polish to prevent drying and preserve fluorescence.

Image analysis

To quantify the **nuclear-to-cytoplasmic** distribution of YAP, fluorescence intensity measurements were performed using ImageJ. DAPI staining was used to generate a nuclear mask. The mean fluorescence intensity of the YAP signal within each nucleus was measured. The cytoplasmic intensity was calculated by subtracting the nuclear signal from the total YAP signal. The nuclear-to-cytoplasmic (n/c) ratio was obtained by dividing the nuclear intensity by the cytoplasmic intensity. Quantification of condensate size and frequency was performed using the **Analyse Particles** function in ImageJ. Airyscan images (lateral resolution of ~120 nm) were adjusted using the “Threshold” function to accurately distinguish condensates from background signal, and the selected threshold range was kept constant throughout the analysis for all conditions. Binary masks were generated, and the “Analyse Particles” function was

used to measure condensate area and frequency. Size filtration was set at 0.05-0.6 μm^2 to measure particles with a diameter of ~250-1000 nm. Co-localization was quantified using **Pearson's correlation coefficient (r)** using the Coloc2 plugin in ImageJ. Cells were manually outlined using the Freehand Selection tool in one fluorescence channel to define the region of interest (ROI). The same ROI was then applied to the corresponding area in the second channel to ensure spatial consistency. **Scratch areas** for wound healing assays were measured using ImageJ. The wound region was outlined using the Freehand Selection tool, and the area was calculated using the "Measure" function under the "Analyze" menu. The remaining wound area at each time point was normalized to the initial (0-hour) scratch area to calculate the percentage of wound closure over time.

Plasmid and gRNA transfection

MDCK and HEK293T cells were transfected with plasmids encoding GFP- or HA-tagged proteins, and/or guide RNAs (gRNAs), when cells reached approximately 60% confluency. Prior to transfection, the culture medium was replaced with fresh antibiotic-free DMEM containing 10% FBS. Transfections were carried out using either polyethylenimine (PEI) or Lipofectamine™ 3000 (Invitrogen #L3000015), depending on the cell type. For MDCK cells, Lipofectamine 3000 was used at a ratio of 3 µL per 1 µg of plasmid DNA. For HEK293T cells, PEI was used at a ratio of 5 µL per 1 µg of plasmid DNA. In 6-well plates, a total of 2 µg DNA was used per well. For transfection in 12-well or 24-well plates, 1 µg DNA per well was used for MDCK cells. DNA and transfection reagents were diluted in DMEM or OptiMEM and incubated at room temperature for 30 minutes to allow complex formation. The transfection mix was then added dropwise to the cells. After 4 hours, the medium was replaced with complete growth medium supplemented with 10% FBS and 1% penicillin–streptomycin. Cells were allowed to grow for 16-24 hours post-transfection before being processed for downstream RNA or protein analyses.

Genomic DNA extraction and Sanger sequencing

Genomic DNA (gDNA) was isolated from cultured cells using the GeneJET Genomic DNA Purification Kit (Thermo Scientific #K0721) according to the manufacturer's instructions. Briefly, cells were harvested and washed twice with PBS. The resulting cell pellet was lysed in a buffer containing proteinase K to degrade cellular proteins, followed by RNase A treatment to eliminate RNA contamination. After complete lysis, ethanol was added to facilitate DNA binding to the silica membrane within the spin column. The lysate was then transferred to the spin column and centrifuged, allowing gDNA to bind to the membrane while contaminants were removed via a series of washes with the supplied buffers. The purified DNA was eluted in RNase-free water. For genotyping of CRISPR-edited loci, PCR amplification was performed using primers flanking the guide RNA target site. Primer sequences were designed using the CRISPOR online tool (http://crispor.tefor.net) to ensure specificity. PCR products were purified and submitted for Sanger sequencing (BioBasic, Singapore).

siRNA transfection

For siRNA transfection, cells were seeded to reach approximately 50% confluency on the day of transfection. Culture medium was replaced with antibiotic-free DMEM containing 10% FBS. siRNA duplexes were transfected at a final concentration of 100 nM using Lipofectamine RNAiMAX (Invitrogen, #13778075). For every 100 nM of siRNA, 3 µL of RNAiMAX was used per well. siRNA and RNAiMAX were diluted in

DMEM only media, incubated for 20–30 minutes at room temperature, and then added dropwise to cells. After 24 hours, the medium was replaced with fresh complete DMEM containing FBS and antibiotics. Cells were harvested for RNA or protein extraction 48 hours post-transfection.

Immunoprecipitation

MDCK or HEK293T cells transfected with GFP-tagged constructs were washed twice with cold phosphate-buffered saline (PBS) and lysed in ice-cold lysis buffer composed of 50 mM Tris-HCl (pH 7.4), 150 mM NaCl, 0.5% Triton X-100, and 1 mM EDTA, supplemented with protease inhibitors (Roche cOmplete™ Mini EDTA-free, #11873580001), and, where indicated, phosphatase inhibitors (PhosSTOP™, Roche). Lysis was performed by incubating the cells with buffer for 10 minutes at 4 °C on a rotating platform. Lysates were clarified by centrifugation at 20,000 × g for 30 minutes at 4 °C. Cleared lysates were incubated with GFP-Trap® Agarose beads (Chromotek, gta-20) at a 1:20 bead-to-lysate dilution in lysis buffer. The bead–lysate mixture was incubated at 4 °C for 2 hours on a rotator to allow binding of GFP-tagged proteins. Following incubation, beads were pelleted by centrifugation at 500 × g for 2 minutes at 4 °C and washed three times with lysis buffer to remove unbound proteins. After the final wash, beads were resuspended in 4× LDS sample buffer (Invitrogen) containing 200 mM dithiothreitol (DTT). Samples were boiled at 96 °C for 5 minutes to elute bound proteins from the beads.

SDS-PAGE and Western Blotting

Protein lysates were mixed with 4× lithium dodecyl sulfate (LDS) sample buffer containing 200 mM dithiothreitol (DTT) and denatured by boiling at 96 °C for 5 minutes. Total protein concentrations were determined using the Bradford assay (Bio-Rad). Equal amounts of protein were separated by SDS-PAGE at 120 V for 1 hour in standard SDS running buffer (25 mM Tris base, 192 mM glycine, 0.2% SDS). Proteins were transferred onto methanol-activated polyvinylidene difluoride (PVDF) membranes (Millipore) in transfer buffer (25 mM Tris, 192 mM glycine, 20% (v/v) methanol) using a wet-transfer system, either overnight at 20 V or for 3 hours at 70 V, both at 4 °C. After transfer, membranes were briefly dehydrated in methanol for 10 seconds and air-dried for 30 minutes to enhance protein retention. Membranes were then blocked and incubated with primary antibodies diluted in blocking buffer containing 2.5% BSA, 0.4% Tween-20, and 1 mM sodium azide. Following three washes with PBS containing 0.1% Tween-20 (PBST), membranes were incubated with horseradish peroxidase (HRP)-conjugated secondary antibodies diluted in 5% non-fat dry milk in PBST. After a further three washes in PBST, blots were developed using an enhanced chemiluminescent (ECL) substrate and imaged using a ChemiDoc imaging system (Bio-Rad).

Transmission Electron Microscopy

EGFP-APEX2 tagged Homer1, Homer2, and Homer3 constructs were transfected into 293T cells using PEI as transfection reagent. Cells were fixed 16-20 hours post-transfection using 2.5% glutaraldehyde (EM grade) in 0.1 M cacodylate buffer, pH 7.4 for 1 hour and further processed for APEX2-TEM as previously described (1, 4). Electron micrographs were captured on a Tecnai T12 TEM (ThermoFisher) operated at 120 kV using an Eagle CCD camera (4k x 4k).

RNA extraction, cDNA synthesis and RT-qPCR

RNA was extracted using PureLink™ RNA Mini Kit (Invitrogen #12183020) following the manufacturer's instructions. To ensure the complete removal of genomic DNA from RNA preparations, 1 µg of RNA was treated with DNase I. Specifically, 1 µL of 10X reaction buffer containing $MgCl_2$, 1 µL of DNase I (TURBO DNA-free™ Kit, Invitrogen #AM1907), and DEPC-treated water to a final volume of 10 µL were incubated at 37°C for 1 hour. After digestion, 1 µL of 50 mM EDTA was added at 65°C for 10 minutes. A total of 1 µL of oligo d(T)15 primer (500 µg/mL; Integrated DNA Technologies), 1 µL of random hexamers (Integrated DNA Technologies), and 1 µL of 10 mM dNTPs. This mixture was incubated at 65°C for 5 minutes, then quickly chilled on ice at 4°C for 10 minutes to allow the primers to anneal. Following annealing, 1 µL of RiboLock RNase inhibitor, 4 µL of 5X SSIV buffer, 1 µL of 100 mM DTT, and 1 µL of reverse transcriptase enzyme (SuperScript IV Reverse Transcriptase, Invitrogen™ #18090010) were added to the annealed oligo mix. The mixture was then incubated at 23°C for 10 minutes, followed by incubation at 53°C for 10 minutes to facilitate the reverse transcription reaction. Finally, the reaction was incubated at 80°C for 10 minutes to inactivate the reverse transcriptase, and the reaction was held at 4°C until further processing. A concentration of 5 ng of cDNA were used for qPCR experiment using SsoAdvanced Universal SYBR green supermix. All qPCR primers are listed in Table S1. Each qPCR reaction was prepared in a total volume of 10 µL, containing 5 µL of SYBR Green Supermix, a mix of 0.5 µL of forward and reverse primer (10 µM), 1 µL of cDNA template (5 ng), and 3.5 µL of nuclease-free water. qPCRs were performed in 96-well plates using a Bio-Rad CFX96 thermocycler. To determine relative fold changes in gene expression, ΔΔCt values were normalised to GAPDH and control conditions (MDCK wild-type cells or control siRNA-transfected cells).

RNA sequencing

RNA sequencing (RNA-seq) of Homer triple knockout (TKO) MDCK cells was performed by NovogeneAIT using an Illumina sequencing platform. Total RNA was extracted and subjected to poly-A selection prior to library preparation. A non-stranded library preparation method was employed, and sequencing was conducted using paired-end reads with a read length of 150 base pairs for each end (2 × 150 bp), resulting in a total of 300 base pairs per fragment. Approximately 80 million reads were generated per sample, consisting of 40 million reads for read 1 and 40 million reads for read 2. NovogeneAIT provided raw sequencing data along with a comprehensive report including quality control (QC) metrics, alignment statistics, and preliminary differential expression results. Downstream analysis of RNA-seq data, including differential gene expression, was performed using R. Heatmaps of RNA-seq data were generated using RStudio to visualize gene expression patterns across different experimental conditions. Raw gene expression data were normalized to eliminate technical variability and log-transformed to stabilize variance across samples. The processed data were structured as a matrix with genes as rows and samples as columns. Heatmaps were generated using the pheatmap package in R, selected for its flexibility and user-friendly interface. The pheatmap function was used to create the heatmaps, with both rows (genes) and columns (samples) hierarchically clustered based on expression similarity using default distance and linkage settings. The top 100 upregulated and downregulated genes identified from the RNA sequencing data were further analyzed for Gene Ontology (GO) enrichment using the ShinyGO 0.8 platform (http://bioinformatics.sdstate.edu/go/). This analysis allowed for the identification of significantly enriched biological processes, molecular functions, and

cellular components associated with the differentially expressed genes. Gene functions were subsequently classified into specific functional groups using the PANTHER classification system (https://www.pantherdb.org/), facilitating a more detailed understanding of the roles these genes play in various biological pathways. Volcano plots were generated in R to visualize differentially expressed genes based on $\log_2$ fold change and adjusted p-values from RNA-seq analysis. Processed differential expression results, including $\log_2$ fold change and adjusted p-values (padj), were imported into R as a data frame. Genes were classified as significantly up- or downregulated based on defined thresholds (e.g., $|\log_2 FC| \geq 1$ and padj < 0.05). The EnhancedVolcano package was used to create the plot. The EnhancedVolcano function was applied, with $\log_2$ fold change on the x-axis and $-\log_{10}$(padj) on the y-axis. Custom colour coding and labelling were used to highlight significantly regulated genes.

Luciferase reporter assays

TEAD (8xGTIIC) and TOPFlash (TCF/LEF) luciferase reporter assays were performed using the Dual-Glo® Luciferase Assay System (Promega, #E2920). For 24-well format experiments, cells were transfected with either 0.4 µg of 8xGTIIC firefly luciferase plasmid (Addgene, #34615) or 0.4 µg of TOPFlash firefly luciferase plasmid (Addgene, #12456), along with 0.04 µg of pRL-TK Renilla luciferase control reporter vector (Promega, #E2241), which served as an internal control for transfection efficiency. After 24–48 hours post-transfection, 100 µL of Dual-Glo® Luciferase Reagent (containing lysis buffer and substrate) was added directly to the wells containing 100 µL of culture medium. The plate was gently mixed and incubated at room temperature for 10 minutes to allow development of the firefly luciferase signal. The mixture was then transferred to a 96-well white opaque plate (Thermo Fisher, #15042), and luminescence was measured using a plate reader. Firefly luciferase activity, representing TEAD or TCF/LEF transcriptional activity, was detected at 560 nm emission. Next, 100 µL of Dual-Glo® Stop & Glo® Reagent was added to each well to simultaneously quench the firefly signal and activate Renilla luciferase. Renilla luminescence, which serves as a normalization control, was measured immediately afterward, at 480 nm. Firefly luciferase values were normalized to corresponding Renilla luciferase values to account for variability in transfection efficiency. Reporter activity was expressed as the ratio of firefly to Renilla luminescence and further normalized to wild-type or control siRNA conditions to provide a relative measure of transcriptional activity.

References

1. B. Tan *et al.*, The Mammalian Crumbs Complex Defines a Distinct Polarity Domain Apical of Epithelial Tight Junctions. *Curr Biol* **30**, 2791-2804 e2796 (2020).
2. E. Martin *et al.*, Bulk exocytosis of large intracellular apical precursor organelles establishes apical domain identity during de novo lumen formation. *bioRxiv* 10.1101/2025.03.06.641813, 2025.2003.2006.641813 (2025).
3. J. Schindelin *et al.*, Fiji: an open-source platform for biological-image analysis. *Nature methods* **9**, 676-682 (2012).
4. A. Ludwig, Selective Visualization of Caveolae by TEM Using APEX2. *Methods in molecular biology* **2169**, 1-10 (2020).

Table S1: qPCR primers, plasmids, and oligonucleotides

REAGENT or RESOURCE			SOURCE	IDENTIFIER
qPCR Primers				
	Forward	Reverse		
Canis Lupus Familiaris				
ADAMDEC	CCACCAGACAGAGAATGGCAA	GCCCCAGGAGATGTTTGCTTT	This study	N/A
ANKRD1	GACTCAGTGAGGCTGAACCG	TTCTGCCAGTGTAGCACCAG	This study	N/A
AXIN2	CTTATCGTGTGGGCAGTAAGA	GTGGGTTCTCGGGAAATGA	This study	N/A
c-MYC	GACTCTGAGGAGGAACAAGAAG	CAGCAGAAGGTGATCCAGAC	This study	N/A
CTGF	TGAAGCGCCTCCCAGTTTTT	TCCCACACGTCTTTGAGCAC	This study	N/A
CYR61	CCAATGACAACGCCTTCTGC	GGATGCACTTTTTGCCCTTCTTA	This study	N/A
DACH1	ACTGCCTCCAGGTTTTCCAC	GCAACTTTCAAGAGCCCCTGT	This study	N/A
DLG2	TGAGATGACCAGAGAGACAGA	CCGTTGGAGAGTCACTGAAG	This study	N/A
FRYL	GGAGCTCTGCCGAAGGTTA	GACATGTTGATGACCTCTGCT	This study	N/A
FZD4	GCCAGCTGCAGTTCTTCCT	AGCGCCTCTTGACTGAAAGA	This study	N/A
GAPDH	TTCCACGGCACAGTCAAG	ACTCAGCACCAGCATCAC	This study	N/A
GNA14	TACCTGACCTACTGCGGCAA	CACACGCAGTCGTTGAGCA	This study	N/A
HAS2	CGGGGGAGATGTCCAGATTTT	ACTGGCAGGCCCTTTCTATG	This study	N/A
Homer1	AGCTGCTCGACTAGCAAAGG	CGCCTGCTGATTCCTGTGAA	This study	N/A
Homer2	GAAAGACCTCCGCAAACAAAG	CCAGGTTCTGATTGTCTCTCTC	This study	N/A
Homer3	GCATCTGACCCAGTTTGCTG	CCTGAGATTTCTCCCTCGCC	This study	N/A
Jun	GACCTTCTACGACGATGCCC	GGTCATGCTCTGCTTCAGGA	This study	N/A
NOTCH3	GTGGATGAATGTGCCTCCCA	TCTCACAGAGCACTCCCAGT	This study	N/A
SOX9	CGAACAGACGCACATTTCCC	CTGGGATTGCCCCGAGTG	This study	N/A
Homo Sapiens				
ANKRD1	TAGCGCCCGAGATAAGTTGC	GTCTGCCTCACAGGCGATAA	This study	N/A
Axin2	CTTATCGTGTGGGCAGTAAGA	GTGGGTTCTCGGGAAATGA	This study	N/A
CTGF	TGAAGCGGCTCCCTGTTTTT	TGAGCACTGGGACCATGAAG	This study	N/A
CYR61	CACCCGGGTTACCAATGACA	GGATGCACTTTTTGCCCTTCTTA	This study	N/A
Has2	AGTGTGGATTATGTACAGGTTTGT	GACATCTCCCCCAACACCTC	This study	N/A
Homer1	AACGGGACAGATGATGAAAGAA	TGTTTGCTGATTGCTGAACTATG	This study	N/A
Homer2	GCAGAGCTCCGAGAAAAGGA	GTCTCTCTCTGCCGCCTCTA	This study	N/A
Homer3	CGCACTCACTGTCTCCTATTT	GCTGTTGATGATGGCCTTG	This study	N/A
GAPDH	TCGGAGTCAACGGATTTGGT	TGAAGGGGTCATTGATGGCA	This study	N/A
PTPN14	CCTCGCAAGACAGCATTT	ATTGGTGACAGTGCCATTTA	This study	N/A
Sox9	CCCCCAACGCCATCTTCAA	CCTGGGATTGCCCCGAGT	This study	N/A
GADD45A	AGGATGGATAAGGTGGGGA	ACGTTATCGGGGTCGACGTT	This study	N/A

cDNA Cloning				
Plasmid	Vector	Primers and cloning strategy	Source	Accession number
GFP PatJ PDZ 1-8	A2E-C1	Primers to amplify PATJ PDZ1-8. RE digested and ligated into A2E-GFP-C1<br>SacII_F: ttttccgcggTTCGGCAAATTGAATATATAG<br>XmaI_R: ttttcccgggTTACCGTTTCCCAACGATGC<br>Insert size: 388-4660	PatJ-myc, provided by Andre Le Bivic	cDNA accession: AJ224747.1
GFP PatJ PDZ 2-7	A2E-C1	Primers to clone PATJ PDZ 2-7 into A2E-GFP-C1 vector via TPCR<br>F:AAGCTTCGAATTCTGCAGTCGACGAGGTTGAGCTCATTAATGA<br>R:CAGGTTCAGGGGGAGGTGTGGTCAATCCTCGTTTCTGATGAAAA<br>Insert size: 742-3979		
GFP PatJ PDZ 3-6	A2E-C1	Primers to clone PATJ PDZ 3-6 into A2E-GFP-C1 vector via TPCR<br>F: AGCTTCGAATTCTGCAGTCGACAATGTTGAGCTTGTGAGAAAAGA<br>R: TCAGGTTCAGGGGGAGGTGTGGTCAGGATGACAAACTCTGAACAA<br>Insert size: 1093-3493		
GFP PatJ PDZ 3-4	A2E-C1	Primers to clone PATJ PDZ 3-4 into A2E-GFP-C1 vector via TPCR<br>F: AGCTCAAGCTTCGAATTCTGCAAATGTTGAGCTTGTGAGAAAAG<br>R: GGTTCAGGGGGAGGTGTGGTTATTCATCATCAAACAACCTCC<br>Insert size: 1081-1930		
GFP PatJ PDZ 5-6	A2E-C1	Primers to clone PATJ PDZ 5-6 into A2E-GFP-C1 vector via TPCR<br>F: GCTCAAGCTTCGAATTCTGCAATTGTTGAACTAGTAAAAGATTG<br>R: CAGGTTCAGGGGGAGGTGTGGCTAGGATGACAAACTCTGAACAA<br>Insert size: 2056-3484		
GFP PatJ PDZ 5-6 HBM / GFP PatJ PDZ 1-8 HBM	A2E-C1	Primer to mutate PATJ PxxF motif in the PDZ5-6 linker by SDM:<br>5'-GAAGCAGCCAAGGGAGCTAGAGAT-3' P812A & F815A		
3xHA-Homer1 / A2E-Homer1	3xHA-C1 / A2E-C1	Primers to amplify Homer1<br>EcoRI_F: ttttgaattctATGGGGGAACAACCTATCTT<br>SacII_R: ttttccgcggTTAGCTGCATTCTAGTAGCT<br>Final vector: APEX2-EGFP-HOMER1 (C1-A2E) and HA-HOMER1<br>(C1-3xHA) Insert size: 1-1065	Source Bioscience	Cat#<br>IRATp970C0118D<br>cDNA accession:<br>BC015502
3xHA-Homer2 / A2E-Homer2	3xHA-C1 / A2E-C1	Primers to amplify Homer2<br>EcoRI_F: ttttgaattctATGGGGGAACAACCTATCTT<br>SacII_R: ttttccgcggCTAGTTATCGGTGC<br>Final vector: APEX2-EGFP-HOMER2 (C1-A2E) and HA-HOMER2<br>(C1-3xHA) Insert size: 1-1032	pRK5-HOMER2	cDNA accession:<br>NM_004839
3xHA-Homer3 / A2E-Homer3	A2E-C1 / A2E-C1	Primers to amplify Homer3<br>EcoRI_F: ttttgaattcgATGTCCACAGCCAGGGAGCA<br>SacII_R: ttttccgcggCTAGGGCGCAGCCTCAG<br>Final vector: APEX2-EGFP-HOMER3 (C1-A2E) and HA-HOMER3<br>(C1-3xHA) Insert size: 1-1086	pRK5-HOMER3	cDNA accession:<br>NM_004838
3xHA-Homer1 EVH1	3xHA-C1	Primers to clone Homer1 (EVH1 domain only) into 3xHA-C1 vector via TPCR<br>F: CAG GTT CAG GGG GAG GTG TGG CTG TCC CGT TGA TAC TTT CCG<br>R:CGGAAAGTATCAACGGGACAGCCACACCTCCCCCTGAACCTG<br>Insert size: 1-446	Source Bioscience	Cat#<br>IRATp970C0118D<br>cDNA accession:<br>BC015502
3xHA-Homer1 CC	3xHA-C1	Primers to clone Homer1 (Coiled coil domain only) into 3xHA-C1 vector via TPCR<br>F: CGAGCTCAAGCTTCGAATTCTGCAAATGCCAAACTCACTGCAGC<br>R: TCAGGTTCAGGGGGAGGTGTGGTTAGCTGCATTCTAGTAGCTTGG		
3xHA-Homer1 G89A	3xHA-C1	Primers to mutate 3xHA Homer1 (G89A) by SDM:<br>F: GGCAAACACCGTTTATGGATTGGCATTCTC<br>R: AAAGATGATGCTCAGAGGAGAATGCCAATC		
mCherry-Homer3	mCherry-C1	SacII and EcoRI-HF were used to cut Homer3 cDNA from GFP-Homer3 and ligated into mCherry-C1 backbone	pRK5-HOMER3	cDNA accession:<br>NM_004838
mCherry-Homer3 Arginine mutant	mCherry-C1	Primers to mutate R-E for three different Arginine residues<br>Arg1-2:<br>F: GTGTACGAGATCATCAGCATC<br>R:GTGTAAGAGCCACCGTAG<br>Arg3:<br>F: GACAGTGAGGCCAACACAGTC<br>R: GCCGACAGTGAGGCCAAC	pRK5-HOMER3	cDNA accession:<br>NM_004838
GFP-FRYL	pEGFP-C1	FRYL was cloned into GFP-C1 backbone using Gibson Assembly HiFi DNA Assembly kit. Insert size : 1-9085<br>GFP C1_F agctttcttgtacGTTGTTAACTTGTTTATTGCAGC<br>GFP C1_R ctgctttttgtaCTTGTACAGCTCGTCCATG<br>FRYL front_F gagctgtacaagTACAAAAAAGCAGAAGGGC<br>FRYL front_R gattgagagagggcAGACAGCAGGTCATAGTG<br>FRYL back_F acctgctgtctGCCCTCTCTCAATCCTCATAC<br>FRYL back_R caagttaacaacGTACAAGAAAGCTGGGCCC	Transomic Technologies, Inc	cDNA accession:<br>BC172707
GFP-FRYL HBM1	pEGFP-C1	Primers to mutate proline and/or phenylalanine in the FRYL PxxF motifs by SDM:<br>P2663A F: CCCCCGCTCTGTCTGC<br>F2666A R: GGTCGCGAGAGGCGAG<br>P2757A F: CTGTCGCTGTGGATGCC<br>F2760A R: AGTGGCGCATTCAGAACAG	Transomic Technologies, Inc	cDNA accession:<br>BC172707
GFP-FRYL HBM2	pEGFP-C1	Primers to mutate proline and/or phenylalanine in the FRYL PxxF motifs by SDM:<br>P2663A F: CCCCCGCTCTGTCTGC<br>F2666A R: GGTCGCGAGAGGCGAG<br>P2757A F: CTGTCGCTGTGGATGCC<br>F2760A R: AGTGGCGCATTCAGAACAG	Transomic Technologies, Inc	cDNA accession:<br>BC172707
GFP-FRYL C-terminus	pEGFP-C1	Primers to clone FRYL C terminus into GFP-C1 vector via TPCR<br>Forward Primer<br>CAAGCTTCGAATTCTGCAGTCGACATGCCAGAGCCTCTAGCTC<br>Reverse Primer<br>AGGTTCAGGGGGAGGTGTGGTCAGAATCCAGTGCTCACCAT	Transomic Technologies, Inc	cDNA accession:<br>BC021803.1
GFP-FRYL C-terminus HBM	pEGFP-C1	Primers to mutate proline and/or phenylalanine in the FRYL PxxF motifs by SDM:<br>PXXF motif 1<br>F: CACCAGCTCTTTCTGCCATCAT<br>R:TGACGCCAGAGGCGACGT<br>PXXF motif 2<br>F: CAGTCGCTGTGGATGCTG<br>R: TGTTGCGCATTCTGAAC	Transomic Technologies, Inc	cDNA accession:<br>BC021803.1
GFP-Furry C-terminus	pEGFP-C1	Primers to clone FRYL C terminus into GFP-C1 vector via TPCR<br>Forward Primer :<br>CGA GCT CAA GCT TCG AAT TCT GCA GAA AGG ATC GCT CAG GTT TG<br>Reverse Primer:<br>AGG TTC AGG GGG AGG TGT GGT CAG AGA CTA GTG CCA GAC AC	Transomic Technologies, Inc	cDNA accession:<br>BC096532
GFP-NDR1	pEGFP-C1	Primers to clone NDR1 into GFP-C1 vector via TPCR<br>F: AGATCTCGAGCTCAAGCTTCGAATTCTATGGCAATGACAGGCTCA<br>R: TTCAGGGGGAGGTGTGGGAGCTATTTTGCTGCTTTCATGTAGGAA<br>Insert size: 1-3595	Source Bioscience	IRAT-100-G2<br>cDNA accession:<br>BC095413.1

Table S2: Materials and Resources

REAGENT or RESOURCE	SOURCE	IDENTIFIER
Antibodies		
Mouse monoclonal anti-β-catenin	BD Biosciences	Cat# 610153; RRID: AB_397555
Rabbit polyclonal anti-FRYL	Invitrogen	Cat# PA5-56644; RRID: AB_2641755
Mouse monoclonal anti-GAPDH	Santa Cruz	Cat# sc-47724; RRID: AB_627678
Mouse monoclonal anti-GFP	Sigma-Aldrich	Cat# 11814460001; RRID: AB_390913
Rat monoclonal anti-HA	Roche	Cat# 11867423001; RRID: AB_390918
Mouse monoclonal anti-HA	Santa Cruz	Cat# sc-7392; RRID: AB_627809
Rabbit polyclonal anti-Homer1	Invitrogen	Cat# PA5-21487; RRID: AB_11155843
Rabbit polyclonal anti-Homer2	Sigma	Cat# HPA040134; RRID: AB_10795272
Mouse monoclonal anti-Homer3	Santa Cruz	Cat# sc-76154; RRID: AB_10988203
Rabbit polyclonal anti-Homer3	Invitrogen	Cat# PA5-59383; RRID: AB_2642508
Mouse monoclonal anti-LATS1	Santa Cruz	Cat# sc-398560; RRID: AB_2909606
Rabbit monoclonal anti-Phospho-LATS1 (Thr1079)	Cell signalling	Cat# 8654S
Mouse monoclonal anti-MOB1a	Santa Cruz	Cat# sc-393212
Rabbit monoclonal anti-Phospho-MOB1 (Thr35)	Cell signalling	Cat# 8699S
Mouse monoclonal anti-NDR1/2	Santa Cruz	Cat# sc-271703; RRID: AB_10714957
Rabbit polyclonal anti-Phospho-STK38 (Thr444, Thr442)	Invitrogen	Cat# PA5-99570; RRID: AB_2818503
Mouse monoclonal anti-Occludin	Invitrogen	Cat# 33-1500; RRID: AB_2533101
Mouse monoclonal anti-Pals1	Santa Cruz	Cat# sc-365411; RRID: AB_10851475
Rabbit polyclonal anti-PatJ	LifeSpan Biosciences	Cat# LS-C410011
Mouse monoclonal anti-YAP	Santa Cruz	Cat# sc-101199; RRID: AB_1131430
Rabbit anti-Phospho-YAP (Ser127)	Cell signalling	Cat# 13008; RRID: AB_2650553
Mouse monoclonal anti-ZO1 (ZO1-1A12)	Thermo Fisher Scientific	Cat# 33-9100; RRID: AB_2533147
Donkey anti-Rabbit IgG (H+L) Alexa Fluor 488	Invitrogen	Cat# A-21206; RRID: AB_2535792
Donkey anti-Mouse IgG (H+L) Alexa Fluor 488	Invitrogen	Cat# A-21202; RRID: AB_141607
Donkey anti-Mouse IgG (H+L) Alexa Fluor 555	Invitrogen	Cat# A-31570; RRID: AB_2536180
Donkey anti-Rabbit IgG (H+L) Alexa Fluor 555	Invitrogen	Cat# A-31572; RRID: AB_162543
Goat anti-Rabbit IgG (H+L) Alexa Fluor 633	Invitrogen	Cat# A-21071; RRID: AB_141419
Goat anti-Mouse IgG (H+L) Alexa Fluor 633	Invitrogen	Cat# A-21052; RRID: AB_2535719
Goat anti-Rat IgG (H+L) Rhodamine	Invitrogen	Cat# 31680; RRID: AB_228357
Goat anti-rabbit IgG (H+L) HRP conjugate	Invitrogen	Cat# A16104; RRID: AB_2534776
Goat anti-mouse IgG (H+L) HRP conjugate	Invitrogen	Cat# A16072; RRID: AB_2534745
Bacterial and virus strains		
NEB® 10-beta Competent *E. coli* (High Efficiency)	NEB	Cat# C3019H
Chemicals, peptides, and recombinant proteins		
Lipofectamine 3000 Reagent	Thermo Fisher Scientific	Cat# L3000015
GFP-Trap Agarose Beads	Chromotek	Cat# gta-20
Paraformaldehyde	Sigma-Aldrich	Cat# 158127
DMEM, high glucose, no glutamine, no calcium	Gibco	Cat# 21068028
D-Sorbitol	Sigma-Aldrich	S1876
VECTASHIELD® Antifade Mounting Medium	Vector Laboratories	Cat# H-1000-10

DAPI	Sigma-Aldrich	Cat# D9542
Phalloidin–Atto 565	Sigma-Aldrich	Cat# 94072
Experimental models: Cell lines		
MDCK-II cells	ECACC	RRID:CVCL_0424; Cat# 00062107
HEK293T cells	ATCC	RRID:CVCL_0063
HCT116	ATCC	RRID:CVCL_0291
DLD-1	ATCC	RRID:CVCL_0248
RKO	ATCC	RRID:CVCL_0504
Oligonucleotides		
siRNA targeting sequence control (non-target)	Santa Cruz	sc-37007
Homer-1 siRNA	Santa Cruz	sc-35581
Homer-2 siRNA	Santa Cruz	sc-42188
Homer-3 siRNA	Santa Cruz	sc-43850
FRYL siRNA : GCCUGGUGAAUAUGUCAUCAAUU	Dharmacon	N/A
gRNA targeting Homer1 (dog): 5'-CACCGTTGCCCGGCTATCGGCCCAC -3'	Integrated DNA Technologies, (IDT)	N/A
gRNA targeting Homer2 (dog): 5'-CACCGATCGTCCGTCCCGTTGACAC-3'	Integrated DNA Technologies, (IDT)	N/A
gRNA targeting Homer3 (dog): 5'-CACCGAGTCGTGCCAACACCGTCTA -3'	Integrated DNA Technologies, (IDT)	N/A
gRNA targeting FRYL (dog): 5'-CACCGAAGATGAATCCTACGAGTAC -3'	Integrated DNA Technologies, (IDT)	N/A
gRNA targeting FRYL (dog): 5' - CACCGAAGATGAATCCTACGAGTAC - 3'	Integrated DNA Technologies, (IDT)	N/A
gRNA targeting PATJ (dog): 5'-CACCGTACACCTAACTCTAGTTCGA-3'	Integrated DNA Technologies, (IDT)	N/A
See Table S2 for qPCR primers	This paper	N/A
Recombinant DNA		
YAP-GFP	Origene	Cat# RC231269L4
8xGTIIC-luciferase	Addgene	Cat# 34615
pRL-TK Renilla Luciferase	Promega	Cat# E2241
M50 Super 8x TOPFlash	Addgene	Cat# 12456
β-catenin-GFP	Addgene	Cat# 71367
GFP PatJ PDZ 1-8	This study	N/A
GFP PatJ PDZ 2-7	This study	N/A
GFP PatJ PDZ 3-6	This study	N/A
GFP PatJ PDZ 3-4	This study	N/A
GFP PatJ PDZ 5-6	This study	N/A
GFP PatJ PDZ 5-6 HBM	This study	N/A
GFP PatJ PDZ 1-8 HBM	This study	N/A
mCherry-PATJ	Pombo-García et al., 2024	N/A
3xHA Homer1	This study	N/A
3xHA Homer1 G89A	This study	N/A
3xHA Homer1 EVH1	This study	N/A
3xHA Homer2	This study	N/A
3xHA Homer3	This study	N/A
A2E-Homer1	Tan et al., 2020	N/A
A2E-Homer2	Tan et al., 2020	N/A

A2E-Homer3	Tan et al., 2020	N/A
mCherry-Homer3	This study	N/A
mCherry-Homer3 R45E, R49E, R84E	This study	N/A
GFP-FRYL	This study	N/A
GFP-FRYL HBM1	This study	N/A
GFP-FRYL HBM2	This study	N/A
GFP-FRYL C-terminus	This study	N/A
GFP-FRYL C-terminus HBM3	This study	N/A
GFP-FRYL C-terminus HBM4	This study	N/A
GFP-Furry	This study	N/A
GFP-NDR1	This study	N/A
See Table S2 for cloning primers	This paper	N/A
Software and algorithms		
ImageJ/Fiji	NIH	https://imagej.nih.gov/ij/
STRING	STRING Consortium	https://string-db.org
RStudio (version 2023.09.1-494)	Posit, PBC	https://posit.co/download/rstudio-desktop
Image Lab Software (version 6.1.0 build 7)	Bio-Rad Laboratories	https://www.bio-rad.com/image-lab
Adobe InDesign (version 2024)	Adobe Inc.	https://www.adobe.com/products/indesign.html
Other		
Transwell filter inserts (6.5 mm)	Corning	Cat# 3413
Glass coverslips (12 mm, #1.5)	Electron Microscopy Sciences (EMS)	Cat# 72230

Supplemental Figures

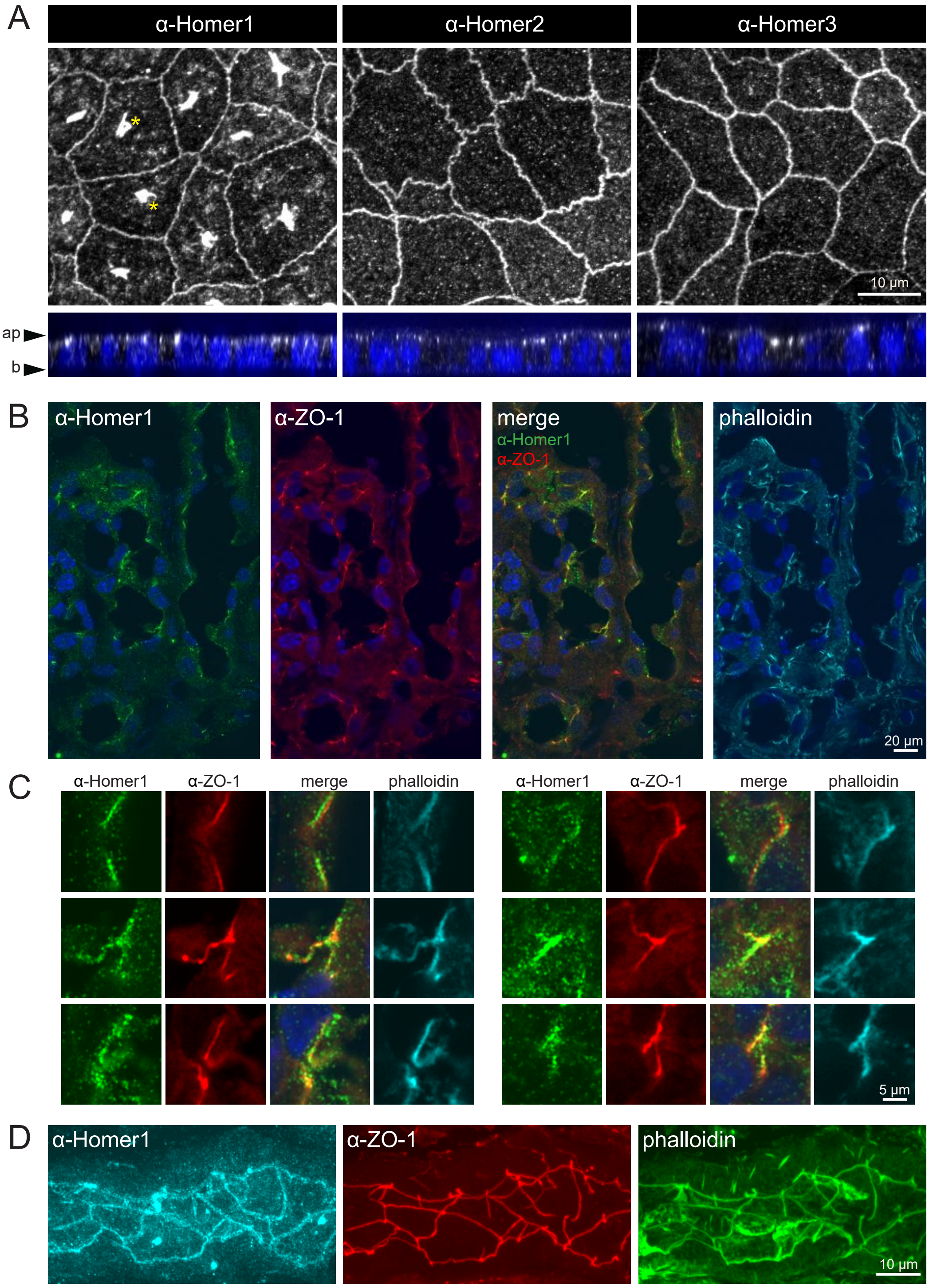

Figure S1: Homers localise to apical cell junctions in renal epithelial cells
(A) Localisation of Homers in MDCK-II cells. Cells were grown to confluency on Transwell filters, fixed and stained with Homer1, Homer2 or Homer3 antibodies. XY (en face) and XZ (side view) projections of confocal z-stacks are shown. Note that anti-Homer1 antibodies produce a non-specific staining of unknown origin in the cytoplasm (yellow asterisks). (B-D) Localisation of Homer1 in mouse kidney cryosections. Sections were stained with antibodies against Homer1 and ZO-1 and counterstained with phalloidin and DAPI. (B) Cross-section through renal tubules. (C)

Magnified views of apical cell junctions based on the images shown in (B). Pearson's r = 0.60 ± 0.08; n=30. (D) En face sections through apical cell junctions in renal epithelial cells.

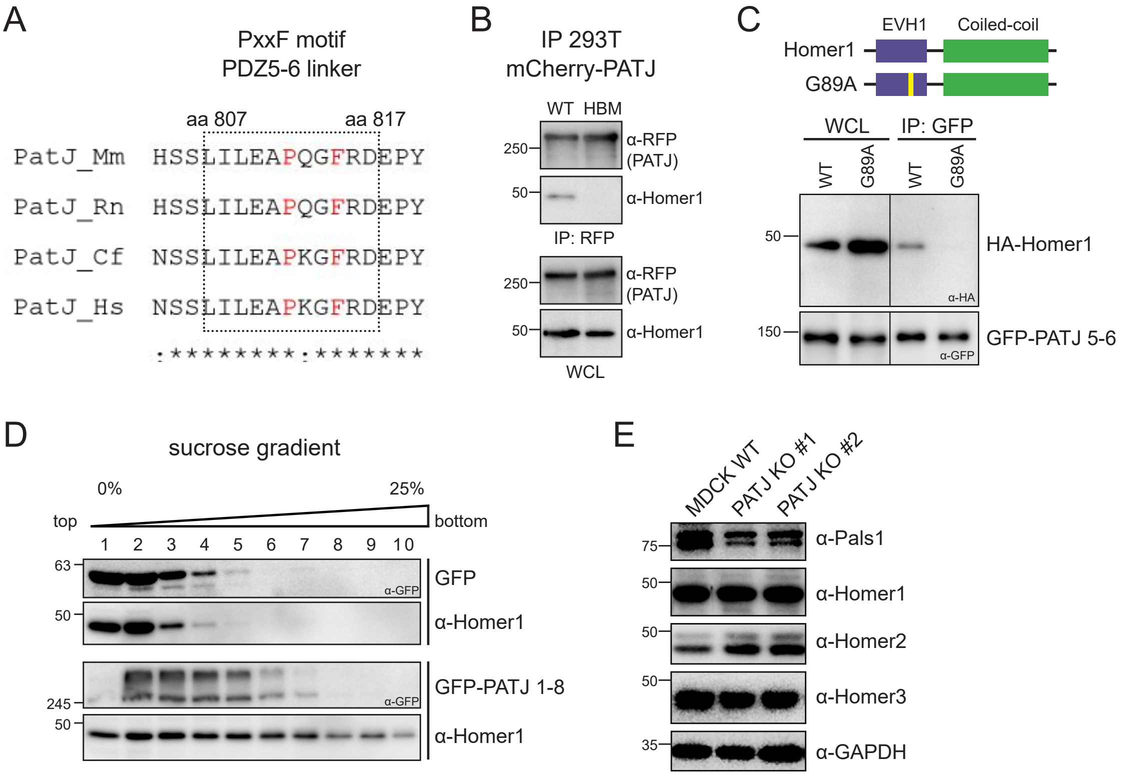

Figure S2: PATJ interacts with Homers

(A) Amino acid sequence alignment of the PxxF motif in PATJ, located between the PDZ5 and PDZ6 domains (aa 807-817). Mm: *Mus musculus*, Rn: *Rattus norvegicus*, Cf: *Canis lupus familiaris*, Hs: *Homo sapiens.* (B) Co-IP assay of full-length wild-type mCherry-PATJ and the corresponding Homer binding mutant (HBM, PKGF to AKGA). (C) Co-IP assay of GFP-PATJ PDZ5-6 with wild-type HA-Homer1 or the EVH1 domain mutant (G89A). (D) Sucrose density gradient centrifugation of 293T cell lysates. Cells were transfected with the A2E-C1 construct (control; top) or the A2E-PATJ PDZ1-8 construct (bottom). Gradient fractions were probed with GFP and Homer1 antibodies. (E) WB analysis of MDCK WT and PATJ KO MDCK cell lysates probed for Pals1, PATJ and Homers.

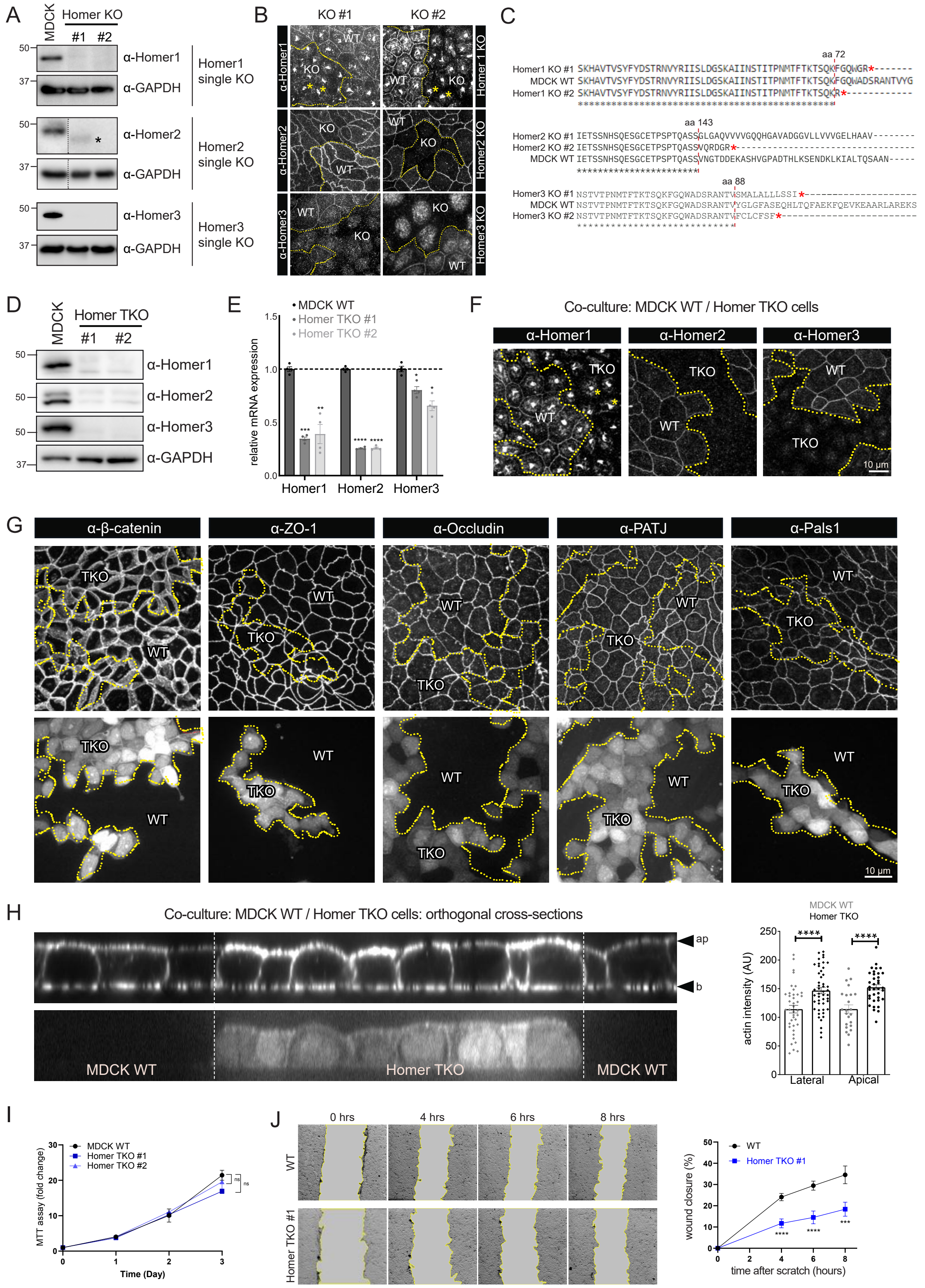

Figure S3: Generation and characterisation of Homer KO cells

(A) WB analysis of single Homer1, Homer2, and Homer3 knockout (KO) MDCK cell lines. Note that a truncated version of Homer2 protein is detected in KO clone #1 (asterisk). (B) MDCK WT cells were co-cultured with single Homer1, Homer2 or

Homer3 KO cells on Transwell filter and stained for the corresponding Homer protein. Dotted lines represent the boundary between the single KO cells and MDCK WT cells. Note that anti-Homer1 antibodies produce a non-specific, non-junctional staining that persists in Homer1 KO cells (yellow asterisks). Note also that Homer2 protein is detected but reduced in Homer2 KO clone #1. (C) Amino acid sequence alignment of single Homer1, Homer2, and Homer3 KO cells compared to MDCK WT at their respective loci. Red asterisks (*) indicate premature stop codons. The red line marks the point at which the sequence between KO and WT genomic DNA diverges. (D) Validation of Homer TKO cells. WB analysis of Homer TKO and MDCK WT cell lysates probed for all three Homer proteins. (E) qPCR analysis of Homer1, Homer2 and Homer3 mRNA expression in Homer TKO cell lines (n=2). (F) Homer TKO and MDCK WT cells were co-cultured on Transwell filters for 10-14 days, fixed and stained for Homer1, Homer2 or Homer3. The dotted lines represent the boundaries between the two cell populations. (G) Homers are not required for the assembly or maintenance of the apical junctional complex. MDCK WT and Homer TKO (GFP positive) cells were co-cultured on Transwell filters, fixed and stained for junctional markers β-catenin, ZO-1, Occludin, PatJ, and Pals1. The dotted lines delineate the boundaries between WT and Homer TKO cell populations. (H) Loss of Homers increases cortical actin levels. MDCK WT and Homer TKO (GFP positive) cells were co-cultured on Transwell filters, fixed and stained with phalloidin. Orthogonal cross-sections through the monolayer and quantification of phalloidin intensity at the apical and lateral membranes are shown (n=2 independent co-cultures). Data is presented as mean ± SEM. Statistical significance is ****$P \leq 0.0001$. (I) MTT cell proliferation assay of MDCK WT and MDCK Homer TKO cells (n=3). (J) Scratch wound assay of confluent WT MDCK cells and Homer TKO cells. Wound areas were quantified using ImageJ and normalised to the 0-hour time point (n=3). Data is presented as mean ± SEM. Statistical analysis was performed using two-way ANOVA. * $P \leq 0.05$, ** $P \leq 0.01$, *** $P \leq 0.001$, **** $P \leq 0.0001$.

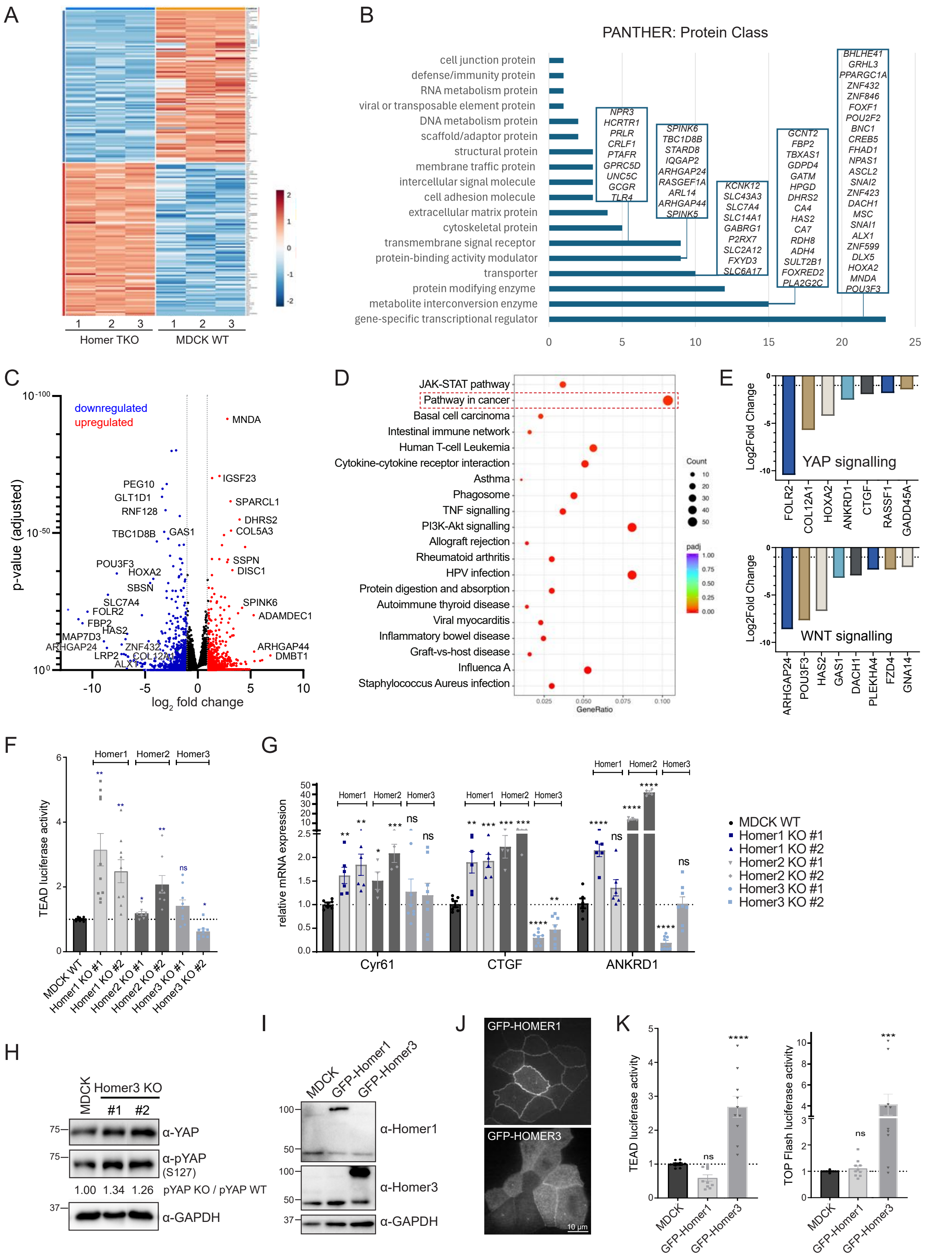

Figure S4: Homers differentially regulate YAP/TEAD activity in MDCK cells
(A) Heatmap showing the top 100 upregulated and top 100 downregulated genes in Homer TKO MDCK cells compared to MDCK WT cells, as identified by RNA-seq analysis (n=3). Colour intensity represents relative expression levels (z-scores), with red indicating upregulation and blue indicating downregulation. See Dataset S1 for a complete list of all differentially expressed genes along with their log2 fold changes

and raw RNA-seq output and a higher resolution figure of the heatmap. (B) The top 100 upregulated and top 100 downregulated genes were classified by PANTHER: Protein Class. Genes in the classification groups are listed in boxes. (C) Volcano plot showing select differentially expressed genes (DEGs) in Homer TKO cells identified by RNA-sequencing. (D) KEGG pathway analysis of DEGs in Homer TKO cells. The y-axis lists significantly enriched pathways, while the x-axis represents the gene ratio. Dot size indicates the number of DEGs involved in each pathway, and colour reflects the adjusted p-value (padj), with red indicating higher statistical significance. (E) Bar graphs showing the log2 fold changes of DEGs associated with the YAP (top) and WNT (bottom) signalling pathways. Genes were selected based on their relevance to each pathway. (F) TEAD luciferase activity in single Homer1, Homer2 and Homer3 KO cells normalised to MDCK WT cells (n=4). (G) qPCR analysis of YAP target gene expression in Homer1, Homer2 and Homer3 KO cells normalised to MDCK WT cells (n=3 or 4). (H) WB analysis of MDCK WT and Homer3 KO cell lysates probed for YAP and pYAP (S127). The pYAP/YAP ratio was quantified by densitometric analysis (n=3). (I) WB analysis of MDCK WT cells and MDCK cells stably transfected with GFP-Homer1 or GFP-Homer3. (J) Representative confocal micrographs of MDCK cells stably transfected with GFP-Homer1 or GFP-Homer3 grown on Transwell filter for 10-14 days. (K) TEAD and TOPFlash luciferase assays of MDCK WT cells and MDCK cells stably transfected with GFP-Homer1 or GFP-Homer3 (n=5). Data is presented as mean ± SEM. Statistical significance is indicated as follows: *P ≤ 0.05; **P ≤ 0.01; ***P ≤ 0.001; ****P ≤ 0.0001.

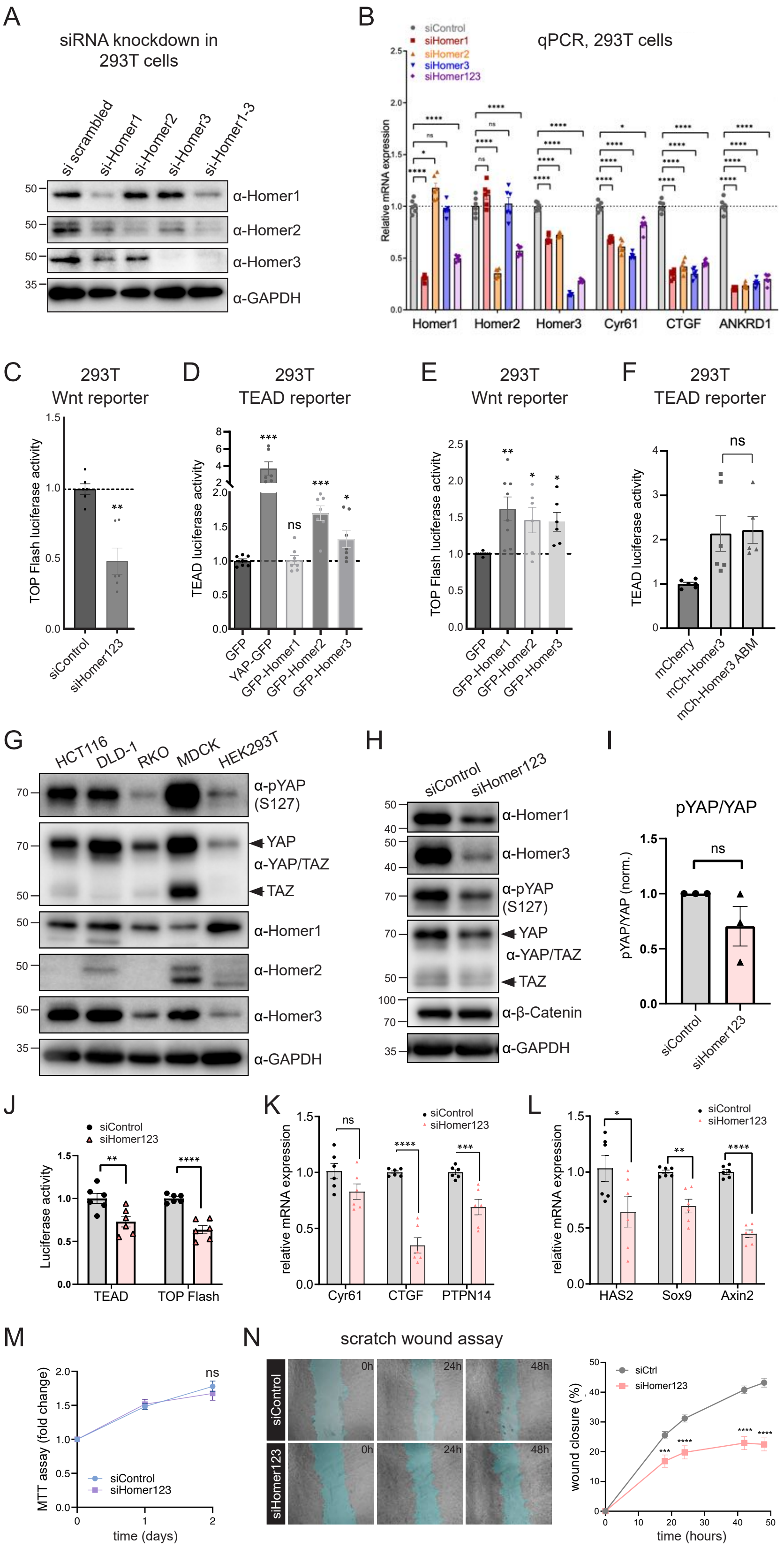

Figure S5: Homers promote YAP/TEAD and Wnt pathway activity in HEK293T and HCT116 cells

(A and B) WB and qPCR analysis of 293T cells transfected with a total of 100 nM siRNA targeting either Homer1, Homer2, or Homer3, or all three Homers

simultaneously (n=3). (C) TOPFlash luciferase activity of 293T cells transfected with a total of 100 nM of control siRNA or a pool of siRNAs targeting all three Homers (n=3). (D) TEAD luciferase assay of 293T cells transfected with GFP or GFP-tagged YAP, Homer1, Homer2 or Homer3 (n=3). (E) TOPFlash luciferase activity in 293T cells transfected with GFP or GFP-tagged Homer1, Homer2, or Homer3 (n=3). (F) TEAD luciferase assay of 293T cells transfected with mCherry-Homer3 or a corresponding actin binding mutant (ABM, R45E, R49E, R84E) of Homer3 (n=3). (G) WB analysis of three colorectal cancer cell lines (HCT116, DLD-1, and RKO) and two non-cancerous cell lines (MDCK-II and HEK293T), probed for the expression of YAP, phosphorylated YAP (S127), Homer1, Homer2, and Homer3. (H) WB analysis of HCT116 cells treated with control siRNA or a pool of siRNAs targeting all three Homers. Cell lysates were collected 48 hours post-transfection and probed for Homer1, Homer3, YAP, pYAP (S127), and β-catenin. (I) Quantification of the pYAP/YAP ratio based on Western blotting in (H) (n=3). (J) TEAD and TOPFlash reporter assays in HCT116 cells transfected with control siRNA or a pool of siRNAs targeting all three Homers (n=3). (K and L) qPCR analysis of YAP target genes (K) and WNT-associated genes (L) in siRNA-transfected HCT116 cells. (M) MTT cell proliferation assay of HCT116 cells transfected with control or Homer siRNA (n=3). (N) Scratch wound assay of siRNA transfected HCT116 cells. Following siRNA transfection, cells were serum-starved for 6 hours prior to scratch induction and maintained in low-serum media. Wound areas were quantified using ImageJ and normalised to the 0-hour time point (n=3). Data is presented as mean ± SEM. Statistical analysis was performed using two-way ANOVA. * $P \leq 0.05$, ** $P \leq 0.01$, *** $P \leq 0.001$, **** $P \leq 0.0001$. All Luciferase assays were statistically analysed using a paired Student’s t-test. All qPCR data were analysed using Two-Way ANOVA Tukey multiple comparison test. Data is presented as mean ± SEM. *$P \leq 0.05$; **$P \leq 0.01$; ***$P \leq 0.001$; ****$P \leq 0.0001$.

A

Rank	Protein	Protein ID	Peptide counts	LFQ intensity
1	FRY like transcription coactivator	A0A5F4C CM7	10;9;9;9;9;9;9; 9;9;9;1;1	42,971,000
2	Homer scaffold protein 1	E2RT37	17;13;9;7	33,328,000
3	PATJ crumbs cell polarity complex	J9JHV1	7;7;7;7;2	32,656,000
4	Stratifin	F1PQ93	7	31,341,000
5	Homer scaffold protein 2	F1PI91	17;14	24,272,000
6	Armadillo repeat containing 2	F1PD78	1	15,010,000
7	Kinesin family member 13B	F1PIS5	3;3;3;3	12,806,000
8	C6orf132	J9PBJ6	1	10,524,000

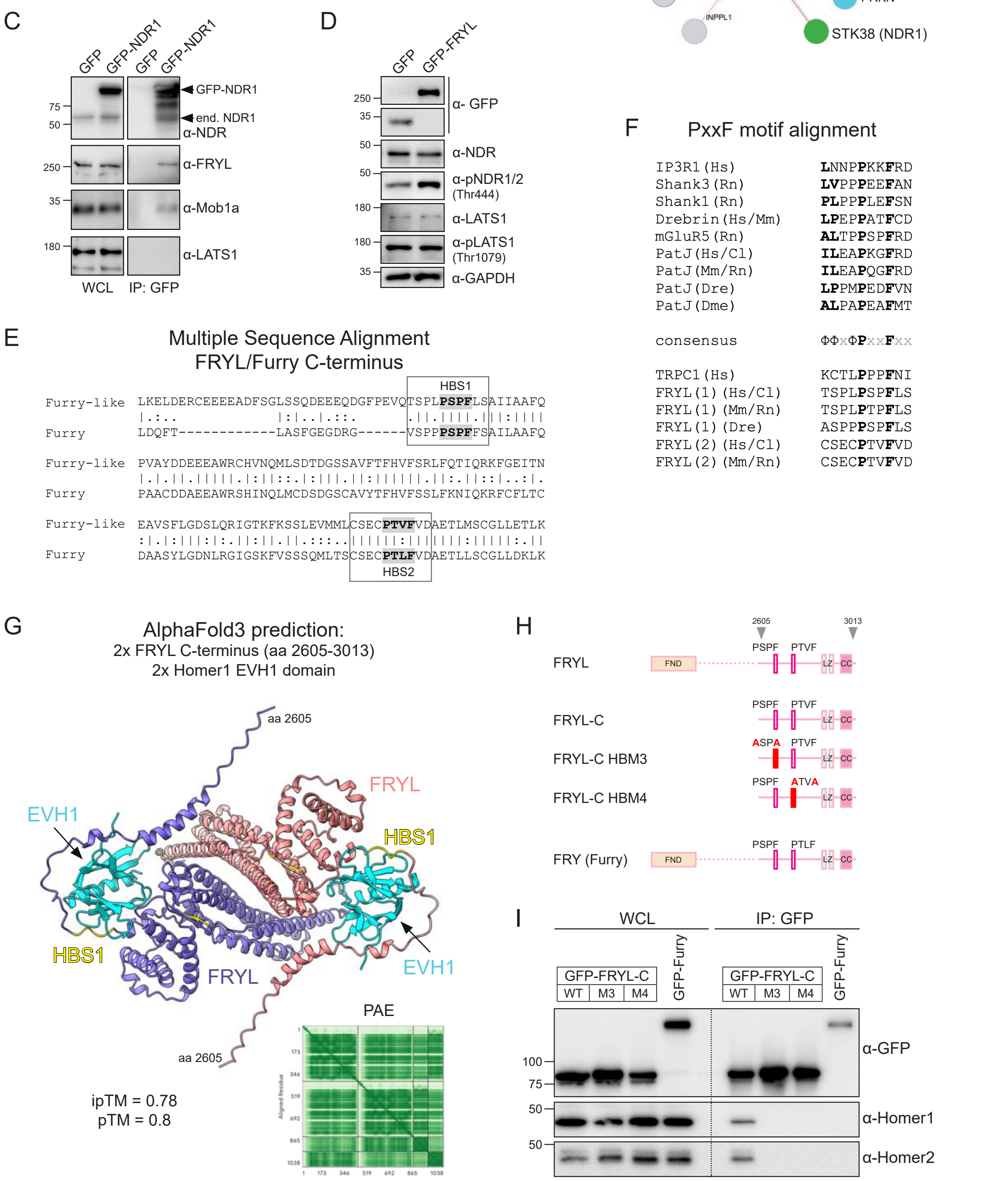

Figure S6: The NDR kinases scaffolding protein FRYL interacts with Homers via two PxxF motifs

(A) IP-MS data of GFP-Homer3 stably overexpressed in MDCK cells. The table shows the top-ranked proteins based on peptide count identified in the MS results. (B) STRING interactome of FRYL based on evidence for physical interactions. (C) IP of

GFP-NDR1 from 293T cell lysates. (D) WB analysis of 293T cells transfected with GFP or GFP-FRYL. (E) Amino acid sequence alignment of the C-terminus of human FRYL and Furry. The two PxxF motifs (Homer binding sites (HBS)) in FRYL are highlighted. (F) Amino acid sequence alignment of PxxF motifs from known Homer EVH1 domain ligands and those identified in PatJ and FRYL. Mm: *Mus musculus*, Rn: *Rattus norvegicus*, Cl: *Canis lupus familiaris*, Hs: *Homo sapiens,* Dre*: Danio rerio*, Dme: *Drosophila melanogaster*. (G) AlphaFold3 prediction of the FRYL C-terminus dimer in complex with two Homer1 EVH1 domains. The HBS1 and HBS2 are highlighted in yellow. HBS1 is predicted to interact with the hydrophobic groove of the EVH1 domains, whilst HBS2 is buried. The Predicted Alignment Error (PAE) plot and the ipTM and pTM values are shown. (H) Cartoon depicting the two Homer binding mutants (HBM) M3 and M4 generated in the context of the FRYL C-terminus (FRYL-C). (I) IP of GFP-Furry, GFP-FRYL-C, and the two GFP-FRYL-C HBMs M3 and M4 from 293T cell lysates. Note that mutation of either of the two PxxF motifs abolishes the interaction with Homers. Furry does not interact with Homers although it contains two PxxF motifs that are similar in primary sequence to those found in FRYL (E).

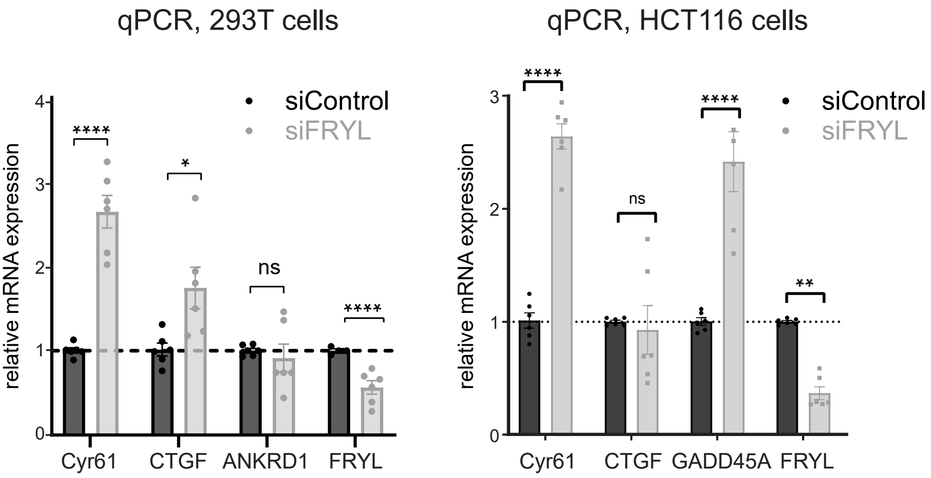

Figure S7: FRYL suppresses YAP target gene transcription in 293T and HCT116 cells

qPCR analysis of canonical YAP target genes in 293T cells (left) and HCT116 cells (right) transfected with control or FRYL siRNA. qPCR data were analysed using Two-Way ANOVA (n=6). Data is presented as mean ± SEM. * P ≤ 0.05, ** P ≤ 0.01, *** P ≤ 0.001, **** P ≤ 0.0001.

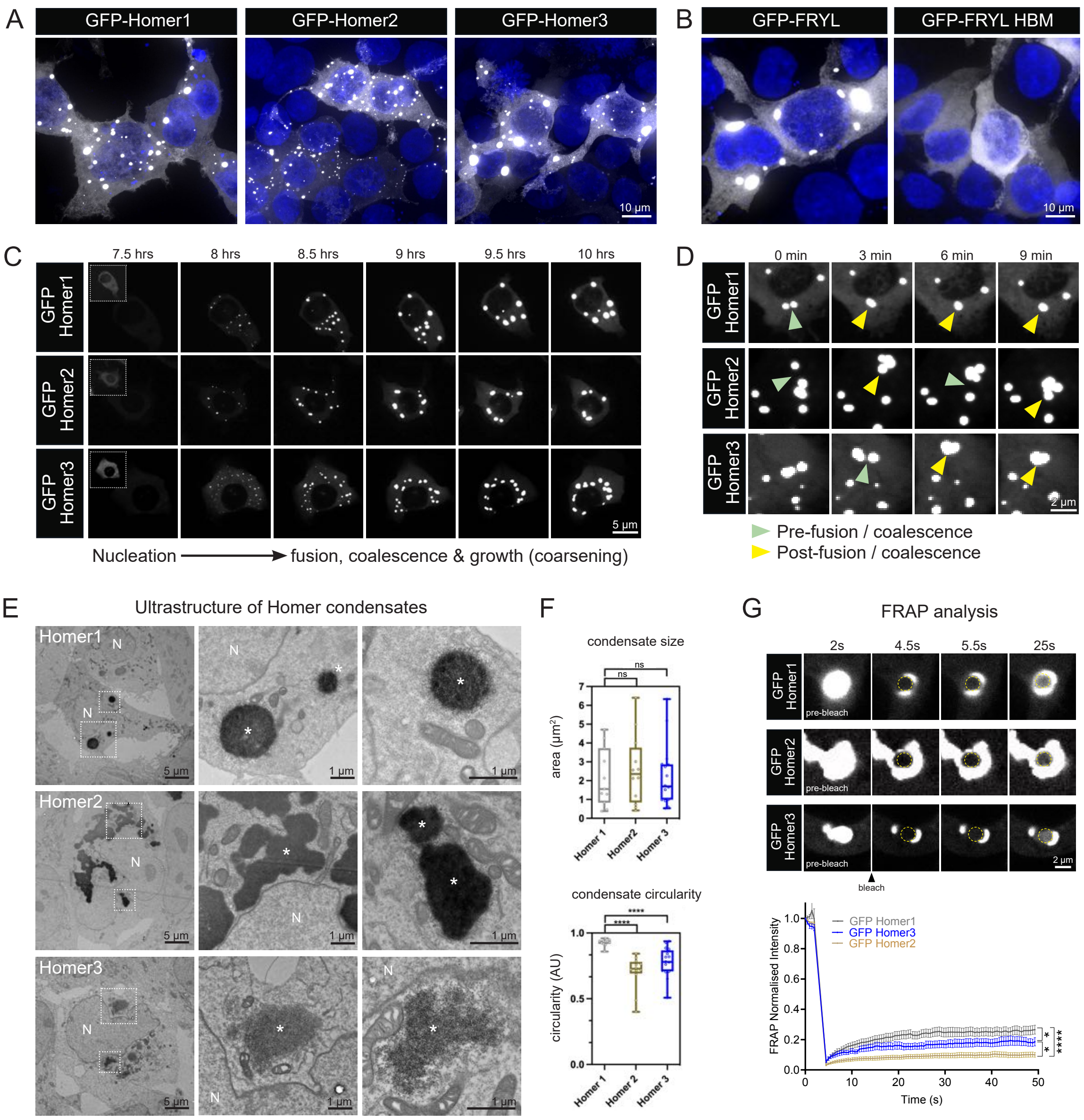

Figure S8: Characterisation of biomolecular condensates induced by transient overexpression of GFP-Homers in 293T cells

(A) Confocal micrographs of 293T cells transiently transfected with GFP-Homer1, GFP-Homer2 or GFP-Homer3. (B) Confocal micrographs of 293T cells transiently transfected with WT GFP-FRYL or GFP-FRYL HBM M2. (C) 293T cells were transfected with GFP-tagged Homer constructs and imaged live 6 hours post-transfection using spinning disk microscopy. Representative time frames illustrating the nucleation, growth and maturation of Homer condensates are shown. The gain in the insets shown at 7.5 hrs was increased to show diffuse cytoplasmic localisation. (D) Live cell imaging illustrating the fusion and coalescence of condensates formed by GFP-tagged Homer1, Homer2, and Homer3. Green arrowheads indicate the prospective site of fusion, yellow arrowheads mark the post-fusion structures. (E) Representative transmission electron micrographs of 293T cells transfected with APEX2-EGFP tagged Homer1, Homer2 or Homer3. EM contrast of Homer condensates was enhanced through APEX2 labeling. Boxed areas are magnified. Note the distinct ultrastructure of Homer condensates. N=nucleus. (F) Quantification of condensate size and circularity based on TEM. ****P ≤ 0.0001 (One-Way ANOVA,

n=12-16 condensates). (G) FRAP analysis of GFP-Homer1, GFP-Homer2 or GFP-Homer3 transiently transfected into 293T cells. Data is presented as mean ± SEM. Statistical analysis was performed using two-way ANOVA. ** $P \leq 0.01$.

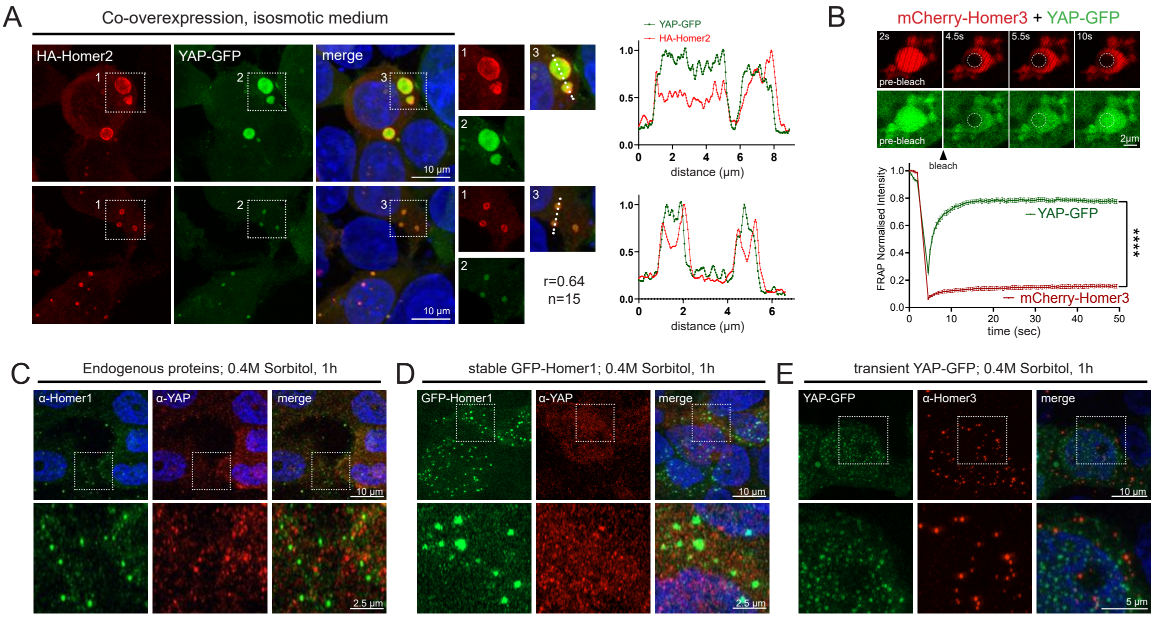

Figure S9: YAP is excluded from Homer condensates formed at or close to endogenous expression levels

(A) 293T cells transiently co-transfected with HA-Homer2 and YAP-GFP. Examples of large (top) and small (bottom) condensates are shown. (B) FRAP analysis of 293T cells co-transfected with mCherry-Homer3 and YAP-GFP (n=31). FRAP data is presented as mean ± SEM. Statistical analysis was performed using two-way ANOVA. ** P ≤ 0.01, *** P ≤ 0.001, **** P ≤ 0.0001. (C) Wild-type 293T cells were exposed to 0.4M sorbitol for 1h, fixed and stained with antibodies against Homer1 and YAP. (D) 293T cells stably transfected with GFP-Homer1 were exposed to 0.4M sorbitol for 1h, fixed and stained with anti-YAP antibodies. (E) 293T cells transiently transfected with YAP-GFP were exposed to 0.4M sorbitol for 1h, fixed and stained with anti-Homer1 antibodies. Images in C-E were acquired in Airyscan mode.

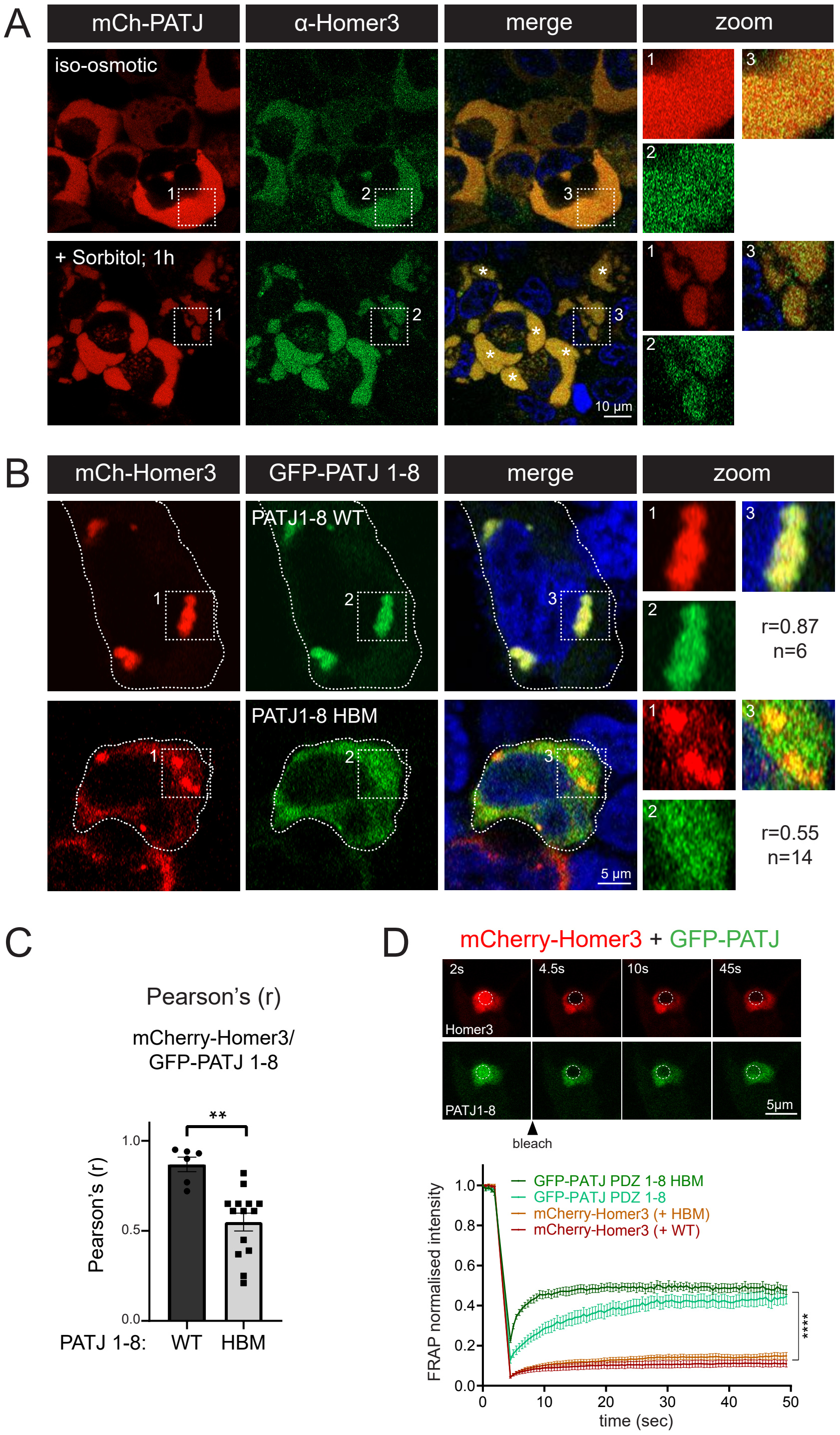

Figure S10: PATJ phase separates with Homers
(A) 293T cells transiently transfected with mCherry-PATJ grown under isosmotic or hyperosmotic conditions (0.4 M sorbitol) for 1 hour were fixed and stained with Homer3 antibodies. (B) 293T cells transiently co-transfected with mCherry-Homer3 and either GFP-PATJ PDZ1-8 or GFP-PATJ PDZ1-8 HBM. (C) Pearson's r co-localization analysis of mCherry-Homer3 with WT and mutant GFP-PATJ PDZ1-8. Data is presented as mean ± SEM. Statistical significance was assessed using Student's t-test (n=15). (D) FRAP analysis of 293T cells co-transfected with mCherry-Homer3 and either GFP-PATJ PDZ1-8 or the corresponding HBM (n=10). FRAP data is presented

as mean ± SEM. Statistical analysis was performed using two-way ANOVA (n=10). * $P \leq 0.05$, ** $P \leq 0.01$, *** $P \leq 0.001$, **** $P \leq 0.0001$.

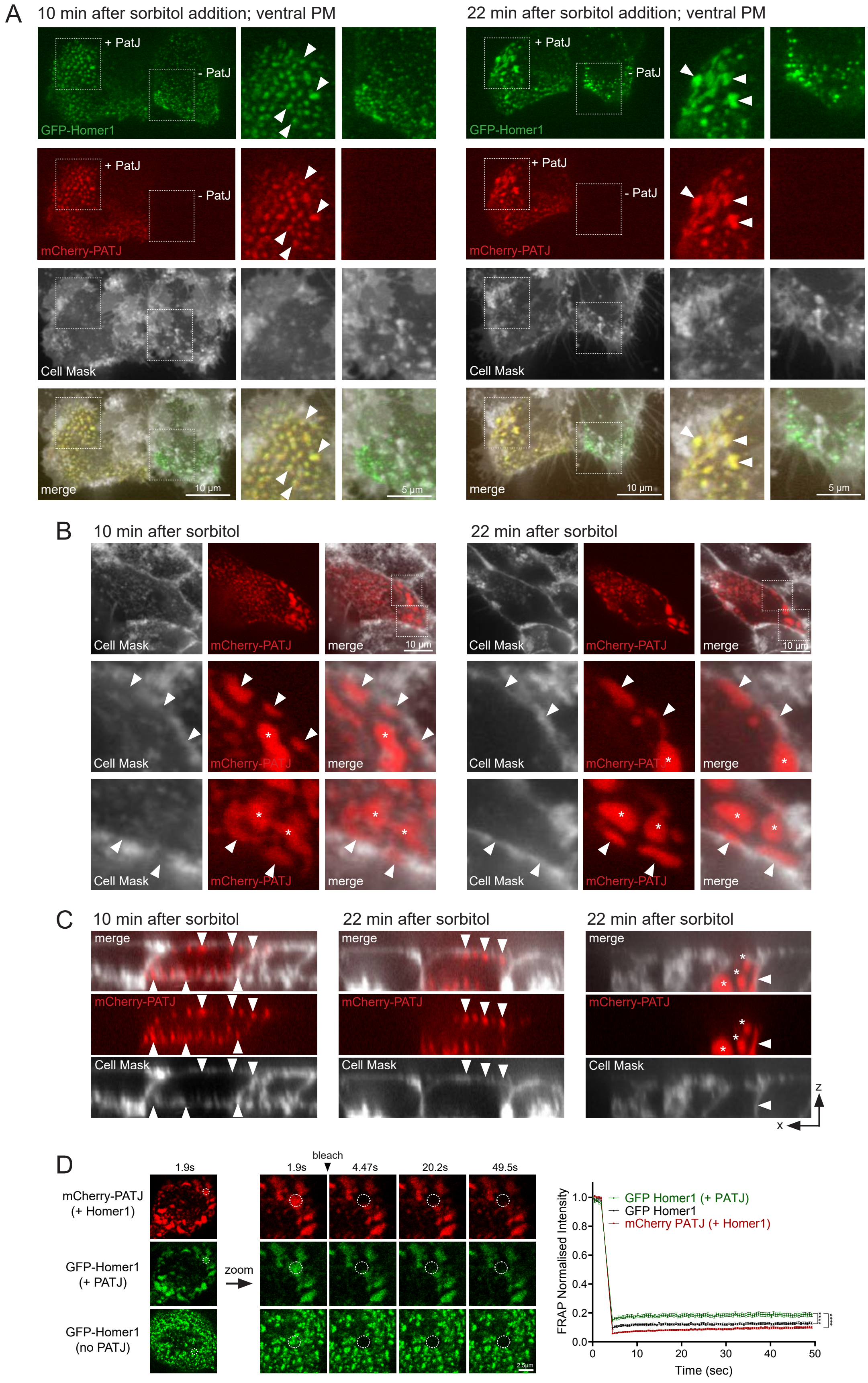

Figure S11: PATJ and Homers phase separate at or near the plasma membrane (A) Live cell confocal microscopy of stable GFP-Homer1 293T cells transiently transfected with mCherry-PATJ and stained with the plasma membrane dye CellMask.

Confocal z-stacks were acquired 10 min after sorbitol addition, using a time interval of 3 min and a z-interval of 200 nm. Confocal optical sections of an mCherry-PatJ-transfected cell (+PatJ) and an untransfected cell (-PatJ) 10 and 22 min after sorbitol addition are shown. Note that PatJ promotes the coalescence of Homers at or near the plasma membrane (arrowheads). (B) Live cell confocal microscopy of 293T cells transiently transfected with mCherry-PATJ and stained with the plasma membrane dye CellMask. Confocal z-stacks were acquired 10 min after sorbitol addition, using a time interval of 3 min and a z-interval of 200 nm. Confocal optical sections 10 and 22 min after sorbitol addition are shown. Arrowheads highlight plasma membrane-associated condensates, asterisks indicate cytoplasmic condensates. (C) Orthogonal X/Z projections of the data shown in (B). Arrowheads highlight membrane-associated condensates, asterisks indicate cytoplasmic condensates. (D) FRAP analysis of stable GFP-Homer1 293T cells transfected with mCherry-PATJ. All FRAP measurements were acquired 5-30 min after sorbitol addition (n=34). FRAP data is presented as mean ± SEM. Statistical analysis was performed using two-way ANOVA (n=34). **** $P \leq 0.0001$.
